# Sex‐ and Time‐Resolved Physiological and Gut Ecosystem Responses to a Food‐Grade Microbiome‐Targeted Intervention in Collegiate Endurance Athletes

**DOI:** 10.1002/advs.77664

**Published:** 2026-10-12

**Authors:** Meng Zhang, Le Ge, Kaiyang Liu, Yanrong Liu, Guolin Zhao, Lai‐Yu Kwok, Zheng Sun, Zhihong Sun, Wenyi Zhang

**Affiliations:** ^1^ Key Laboratory of Dairy Biotechnology and Engineering Ministry of Education Inner Mongolia Agricultural University Hohhot China; ^2^ Key Laboratory of Dairy Products Processing Ministry of Agriculture and Rural Affairs Inner Mongolia Agricultural University Hohhot China; ^3^ Inner Mongolia Key Laboratory of Dairy Biotechnology and Engineering Inner Mongolia Agricultural University Hohhot China; ^4^ Department of Physical Education Inner Mongolia Agricultural University Hohhot China; ^5^ Channing Division of Network Medicine Brigham and Women's Hospital and Harvard Medical School Boston MA USA

**Keywords:** collegiate endurance athletes, digital phenotyping, gut microbiome, indirect‐effect analysis, metabolomics, microbiome‐targeted intervention, sex differences

## Abstract

Endurance athletes experience sustained physiological demands during high‐volume training, yet evidence for microbiome‐targeted interventions in real‐world settings remains limited. We conducted a two‐stage, randomized, double‐blind, placebo‐controlled trial in 150 athletes allocated 2:1 to combined probiotics‐yeast extract (CPYE) or placebo for 6 weeks; 44 completers confirmed for the 12th Inner Mongolia Autonomous Region University Games entered an exploratory 6‐week extension with intensified training. Integrating wearable‐derived phenotyping, field‐based cardiorespiratory indices, clinical assessments, fecal metagenomics, and metabolomics, we found that during the randomized short‐term phase, CPYE was associated with changes in device‐estimated maximal oxygen uptake, exercise heart‐rate indices, lactate, and redox biomarkers versus placebo. Exploratory sex‐stratified analyses suggested heterogeneous response patterns, while the selected long‐term extension indicated time‐dependent changes requiring confirmation. Multi‐omics identified sex‐ and phase‐associated differences in microbial taxa, functions, metabolites, and network topology. Indirect‐effect models nominated candidate features (*rmlD*, *PISD*‐linked phosphatidylethanolamine pathways, *Bacteroides ovatus*, and folate‐related functions) as potential indirect correlates of host outcomes. These findings support further evaluation of CPYE‐associated responses in collegiate endurance athletes; subgroup, long‐term, network, and indirect‐effect results are hypothesis‐generating.

## Graphical abstract

In collegiate endurance athletes, a 6‐week randomized CPYE intervention yielded time‐ and sex‐dependent shifts in field‐based cardiorespiratory, metabolic, and redox indices, accompanied by changes in gut multi‐omics profiles. An exploratory extension during intensified training generates hypotheses warranting future validation.

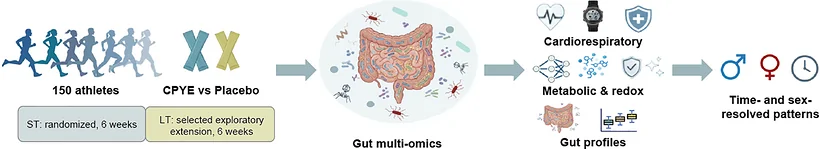

## 1 Introduction

Endurance athletes face sustained physiological demands, and even modest changes in performance, recovery, and resilience may be relevant in competitive settings. While periodized training, optimized nutrition, and advanced recovery protocols have substantially refined athletic preparation, the potential for further gains within these established frameworks is increasingly constrained [1, 2]. This has intensified the search for safe, regulation‐compliant interventions that can be seamlessly integrated into existing regimens to support physiological adaptation and recovery under sustained training, without compromising long‐term well‐being [3].

The gut microbiome represents a critical interface linking diet, exercise stress, and systemic physiology. Growing evidence implicates gut microbial composition and function in modulating endurance performance, metabolic flexibility, redox homeostasis, and gastrointestinal tolerance during intense exercise [4, 5, 6, 7]. Probiotic interventions have shown modest but promising effects in reducing exercise‐associated gastrointestinal distress, lowering the incidence of upper respiratory infections, and improving select performance metrics [3, 6, 8]. Mechanistic studies in animal models further demonstrate that specific microbes, such as lactate‐utilizing *Veillonella*, can convert exercise‐derived lactate into bioactive short‐chain fatty acids that enhance muscle energetics and endurance [9]. Complementarily, yeast extracts, rich in nucleotides, free amino acids, and small peptides, support intestinal barrier integrity and serve as substrates for commensal bacteria, thereby reinforcing mucosal immunity and microbial stability [10, 11, 12]. Together, these findings support the hypothesis that a rationally formulated combination of probiotics and yeast extract could promote complementary microbe‐substrate interactions within the gut ecosystem, with potential consequences for host metabolic and immune responses. We thus selected a combined probiotics‐yeast extract (CPYE) formulation before trial initiation based on prior experimental evidence and our formulation‐development work: our previous animal trial supported combining *Lacticaseibacillus paracasei* Zhang with yeast polypeptides [12], while published evidence on *Lactobacillus*‐*Bifidobacterium* supplementation in exercise settings and prior screening informed the inclusion of *Bifidobacterium animalis* subsp. *lactis* ProSci‐246 [13].

Despite this conceptual promise, the current evidence remains fragmented. Most human trials rely on narrow outcome sets, such as single‐time point performance tests, limited biomarker panels, or low‐resolution microbiome profiles, and rarely integrate deep physiological phenotyping with high‐resolution multi‐omics [3, 14, 15]. Critically, both athletic adaptation and microbiome dynamics are inherently time‐dependent, yet few studies have systematically compared short‐term versus sustained intervention effects within the same cohort [16, 17]. Moreover, sex is a fundamental biological variable in exercise physiology: men and women differ markedly in substrate utilization, hormonal regulation, body composition, and training responsiveness [8, 18]. Yet in microbiome research, sex is often treated as a statistical nuisance rather than a primary axis of biological variation [19, 20, 21]. Finally, while multi‐omics approaches have begun to map associations between microbial features and host traits, statistically supported and biologically interpretable links between microbiome‐targeted interventions and improvements in physical function remain limited in humans.

To address these gaps, we conducted a two‐stage, randomized, double‐blind, placebo‐controlled trial to evaluate whether a food‐grade microbiome‐targeted intervention is associated with changes in field‐based physiological outcomes and gut ecosystem features that vary by sex and intervention duration. We enrolled 150 collegiate endurance athletes undergoing high‐volume training and evaluated a CPYE formulation over a 6‐week short‐term (ST) phase, followed by a selected 6‐week long‐term (LT) extension among ST completers confirmed to compete in the 12th Inner Mongolia Autonomous Region University Games; these participants underwent intensified training during the extension. We assessed 45 complementary metrics, including device‐estimated maximal oxygen uptake (VO_2_ max; a wearable‐derived, field‐based cardiorespiratory index), exercise heart‐rate dynamics, post‐exercise blood lactate (hereafter referred to as “lactate”), fasting glucose concentration, bioelectrical impedance‐derived body‐composition estimates, serum antioxidant and metabolic markers, and continuous wearable‐derived sleep, heart‐rate, and stress metrics (all abbreviations, definitions, and units are provided in Table S1). Concurrently, serial fecal samples were analyzed via shotgun metagenomics and untargeted metabolomics, enabling longitudinal characterization of bacterial, fungal, and phage communities; microbial functional pathways; and fecal metabolite networks. This integrated design allowed us to estimate CPYE‐associated changes in field‐based physiological outcomes during the randomized ST phase and to explore candidate microbial and metabolic pathways associated with sex‐ and time‐resolved response patterns (Figure 1). Sex‐stratified, LT, multi‐omics, network, and indirect‐effect analyses are presented as exploratory and hypothesis‐generating.

**FIGURE 1:**
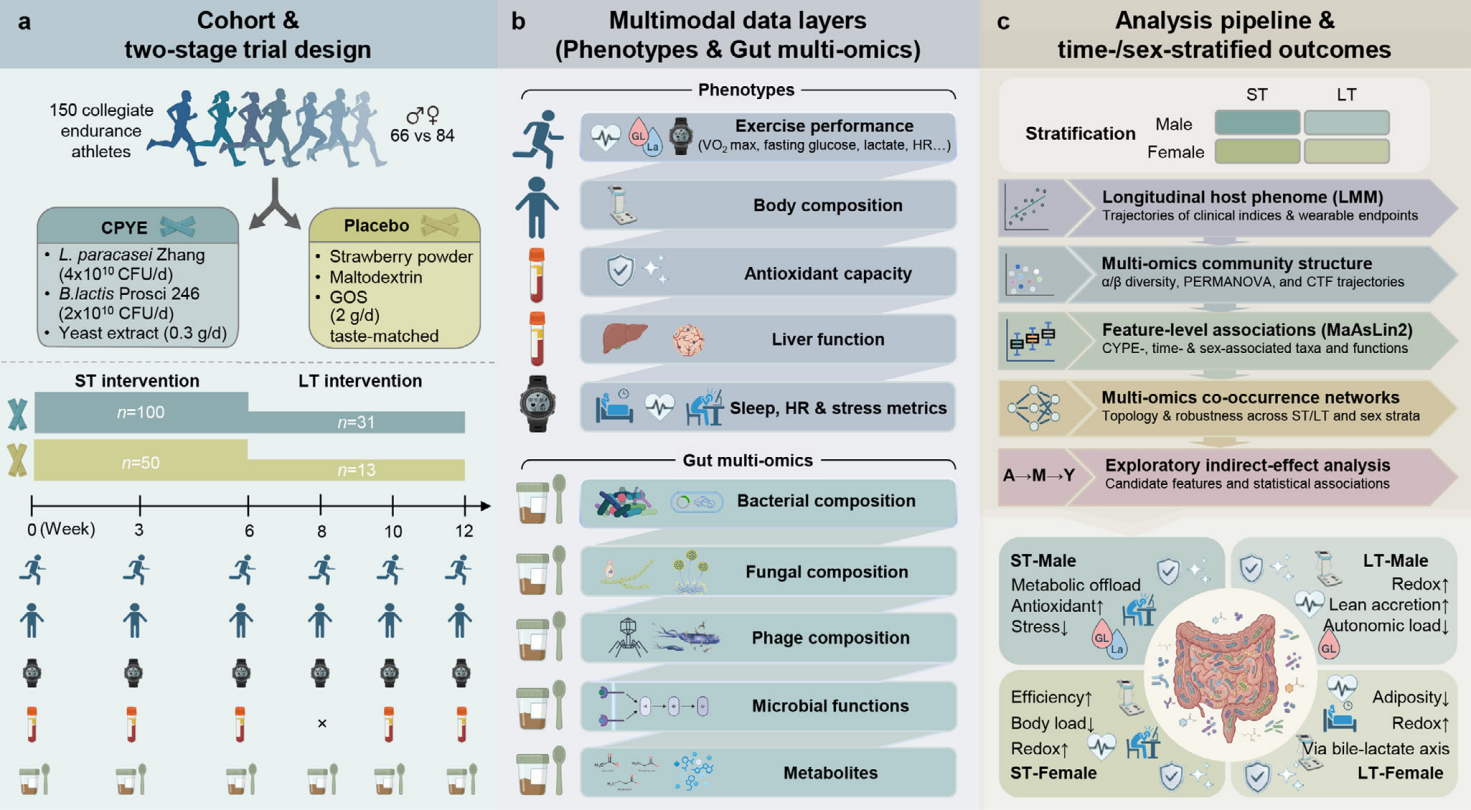
Cohort design, multimodal phenotyping, and analytical framework. (a) Schematic of the 12‐week, two‐stage, randomized, double‐blind, placebo‐controlled trial in 150 collegiate endurance athletes. Participants were randomized 2:1 to receive a combined probiotics‐yeast extract (CPYE; *Lacticaseibacillus paracasei* Zhang, 4 × 10^10^ CFU/day; *Bifidobacterium animalis* subsp. *lactis* ProSci‐246, 2 × 10^10^ CFU/day; yeast extract, 0.3 g/day; total 2 g/day) or a matrix‐matched placebo (identical proportion of galacto‐oligosaccharides and strawberry powder; 2 g/day total). The 6‐week randomized short‐term phase (ST; Weeks 0–6; CPYE *n* = 100, placebo *n* = 50) was followed by a selected long‐term extension (LT; Weeks 6–12) among ST completers competing in the 12th Inner Mongolia Autonomous Region University Games (CPYE *n* = 31, placebo *n* = 13), who underwent intensified training. Visits occurred at Weeks 0, 3, 6, 8, 10, and 12 with assessments of field‐based cardiorespiratory indices, body composition, sleep, heart rate (HR), stress monitoring, and biospecimen collection. (b) Longitudinal phenotypic and gut multi‐omics layers, including device‐estimated maximal oxygen uptake (VO_2_ max), exercise HR dynamics, fasting glucose, post‐exercise blood lactate (lactate), body composition, serum antioxidant and biochemical markers, liver function, and sleep/HR/stress metrics, and fecal bacterial, fungal, phage, functional potential, and metabolite profiles. (c) Analysis workflow stratified by phase (ST/LT) and sex: host phenotypes (linear mixed‐effects models [LMMs]); gut multi‐omics (α/β diversity, permutational multivariate analysis of variance [PERMANOVA], and compositional tensor factorization [CTF] analyses); feature‐level associations (Microbiome Multivariable Association with Linear Models [MaAsLin2]); and multi‐omics co‐occurrence networks (descriptive topology and simulated‐robustness metrics). Exploratory indirect‐effect analysis evaluated candidate microbiome‐metabolite associations with field‐based physiological outcomes. The central gut schematic (bottom) summarizes exploratory response patterns by phase‐sex stratum (ST‐male, ST‐female, LT‐male, LT‐female). Additional abbreviations are defined in Table S1.

## 2 Results

### 2.1 Study Design and Baseline Cohort Characteristics

We conducted a two‐stage, randomized, double‐blind, placebo‐controlled trial to evaluate CPYE‐associated changes in field‐based physiological outcomes and gut ecosystem features, comprising a 6‐week randomized ST phase followed by a post‐randomization, selected 6‐week LT extension. Of 162 collegiate athlete volunteers assessed for eligibility, 12 were excluded, and the remaining 150 were randomized in a 2:1 ratio to CPYE (*n* = 100) or placebo (*n* = 50; Figure S1). One participant in the CPYE group withdrew on Day 17 because of an injury unrelated to the intervention and contributed baseline data only. Thus, 149 participants completed the ST phase (CPYE, *n* = 99; placebo, *n* = 50). Following the ST phase, 44 participants retained their original treatment allocation and entered the LT phase (CPYE, *n* = 31; placebo, *n* = 13). Eligibility for the LT phase required completion of the ST phase and confirmed participation in the 12th Inner Mongolia Autonomous Region University Games; these participants underwent intensified training during Weeks 6–12. The remaining 105 ST‐phase completers did not enter the LT phase (CPYE, *n* = 68; placebo, *n* = 37). All 44 LT participants completed the Week‐12 follow‐up, although the availability of specific phenotypic measurements and biospecimens varied by assessment domain (Figure 1a and Figure S2). Because LT entry was determined after randomization and the extension cohort was smaller, all LT and LT‐by‐sex analyses were considered exploratory and hypothesis‐generating.

Baseline characteristics of the 150 randomized participants are summarized in Table 1. Most demographic, anthropometric, body‐composition, and field‐based physiological characteristics showed small absolute standardized differences between the randomized groups, and sex distribution was identical. The largest baseline standardized mean differences (SMDs) among reported characteristics were observed for fasting glucose (absolute SMD = 0.41) and total antioxidant capacity (T‐AOC, absolute SMD = 0.83). Subsequent longitudinal phenotypic analyses evaluated within‐participant changes from baseline rather than unadjusted post‐intervention values; thus, reported treatment comparisons reflected differential changes relative to each participant's own starting level. Baseline comparisons were descriptive and were not interpreted as significance tests of randomization success. Characteristics of the selected LT cohort and comparisons between LT entrants and non‐entrants are reported separately in Table S2.

**TABLE 1:** Baseline characteristics of 150 randomized participants.

Category	Characteristic	Overall (*n* = 150)	CPYE (*n* = 100)	Placebo (*n* = 50)	Absolute SMD
Demographic characteristics	Age, years	20.3 ± 1.3	20.3 ± 1.3	20.4 ± 1.2	0.07
	Sex, *n* (%)				0.00
	*Female*	84 (56.0)	56 (56.0)	28 (56.0)	
	*Male*	66 (44.0)	44 (44.0)	22 (44.0)	
	Ethnicity, *n* (%)				0.16
	*Han*	70 (46.7)	47 (47.0)	23 (46.0)	
	*Mongolian*	74 (49.3)	48 (48.0)	26 (52.0)	
	*Other*	6 (4.0)	5 (5.0)	1 (2.0)	
Anthropometric and body‐composition characteristics	Body mass index, kg/m^2^	22.0 ± 3.7	21.8 ± 3.6	22.3 ± 3.9	0.13
	Percentage body fat, %	23.1 ± 7.1	22.8 ± 6.8	23.8 ± 7.6	0.13
	Body weight, kg	68.0 ± 16.0	67.5 ± 15.3	68.9 ± 17.4	0.09
	Fat‐free mass, kg	52.0 ± 12.0	51.9 ± 11.5	52.3 ± 13.0	0.03
Field‐based physiological indices	Device‐estimated VO_2_ max, mL/kg/min	49.3 ± 6.0	49.2 ± 5.9	49.6 ± 6.3	0.07
	ExHRave, bpm	155.4 ± 13.6	156.4 ± 13.8	153.4 ± 12.9	0.23
	ExHRmax, bpm	176.0 ± 14.3	176.2 ± 13.9	175.4 ± 15.2	0.06
	Post‐exercise blood lactate, mmol/L	4.9 ± 3.4	5.2 ± 3.5	4.4 ± 3.4	0.24
Metabolic and redox‐related measures	Fasting glucose, mmol/L	5.7 ± 0.9	5.9 ± 0.9	5.5 ± 0.7	0.41
	T‐AOC, µmol/mL	0.131 ± 0.027	0.124 ± 0.028	0.145 ± 0.017	0.83
	GPX activity, U/mL	98.4 ± 48.7	99.4 ± 56.5	96.3 ± 27.4	0.06
	MDA, nmol/mL	2.32 ± 1.60	2.37 ± 1.65	2.23 ± 1.50	0.09

*Note*: Standardized mean differences are reported as absolute values. Continuous SMDs were calculated using the sample‐size pooled SD, whereas binary SMDs used a standardized proportion difference. For the multicategory ethnicity variable, a generalized Mahalanobis‐distance SMD was applied. Only observed baseline data were used.

Abbreviations: CPYE, combined probiotics‐yeast extract; ExHRave, average exercise heart rate; ExHRmax, maximal exercise heart rate; GPX, glutathione peroxidase; MDA, malondialdehyde; SMD, standardized mean difference; T‐AOC, total antioxidant capacity.

Selected baseline demographic characteristics were descriptively similar between randomized groups. In the ST cohort, sex distribution did not differ (CPYE: 44 male, 56 female; placebo: 22 male, 28 female; χ^2^ test, *p* = 1.00). Mean age (20.29 ± 1.29 vs. 20.38 ± 1.18 years; *p* = 0.606) and body mass index (BMI, 21.80 ± 3.64 vs. 22.27 ± 3.88 kg/m^2^; *p* = 0.914) were comparable, as were ethnic distributions (Han/Mongolian/other: CPYE, 47/48/5; placebo, 23/26/1; χ^2^ test, *p* = 0.649). The smaller, selected LT cohort had the following demographic characteristics (CPYE: 8 male, 23 female; age 20.13 ± 1.10 years; BMI 20.88 ± 3.34 kg/m^2^; placebo: 6 male, 7 female; age 20.69 ± 1.64 years; BMI 23.53 ± 4.77 kg/m^2^). To characterize observable selection into the extension, we compared the 44 LT entrants with the 105 ST completers who did not enter LT using original randomized treatment, sex, age, BMI, and eight selected key physiological measures at baseline and Week 6, which marked the start of the LT extension (Table S2, Panel A). At baseline, the largest absolute SMDs among these characteristics were observed for average exercise heart rate (SMD = 0.46), female sex (SMD = 0.37), fasting glucose (SMD = 0.35), device‐estimated VO_2_ max (SMD = −0.35), and glutathione peroxidase (GPX) activity (SMD = ‐0.25). At Week 6, however, the eight selected physiological measures showed smaller observable differences, with all absolute SMDs < 0.20. Thus, measured physiological profiles were more similar at the start of LT, although post‐randomization selection related to sex and other measured or unmeasured characteristics could not be excluded. These findings informed the covariate‐adjusted LT sensitivity analyses described below.

Across both phases, we generated a dense, longitudinal phenotypic atlas by integrating multimodal data, including device‐estimated VO_2_ max, average and maximal exercise heart rates (ExHRave, ExHRmax), lactate, fasting glucose, serum antioxidants, and lipid peroxidation markers used to gauge recovery from exercise stress (T‐AOC; GPX activity; malondialdehyde, MDA concentration), liver function, body composition, and fecal multi‐omics at predefined time points (Figure 1b and Figure S2).

### 2.2 CPYE‐Associated Changes in Field‐Based Cardiorespiratory, Metabolic, and Redox Indices

To characterize CPYE‐placebo differences while accounting for inter‐individual variability, we modeled within‐participant changes (Δ = follow‐up minus baseline) for all clinical and biochemical outcomes using linear mixed‐effects models (LMMs). Fixed effects included age, BMI, body fat percentage (PBF), and ethnicity, with participant modeled as a random intercept; treatment contrasts represent CPYE minus placebo. Analyses were stratified by phase (ST and LT; Figure 2).

**FIGURE 2:**
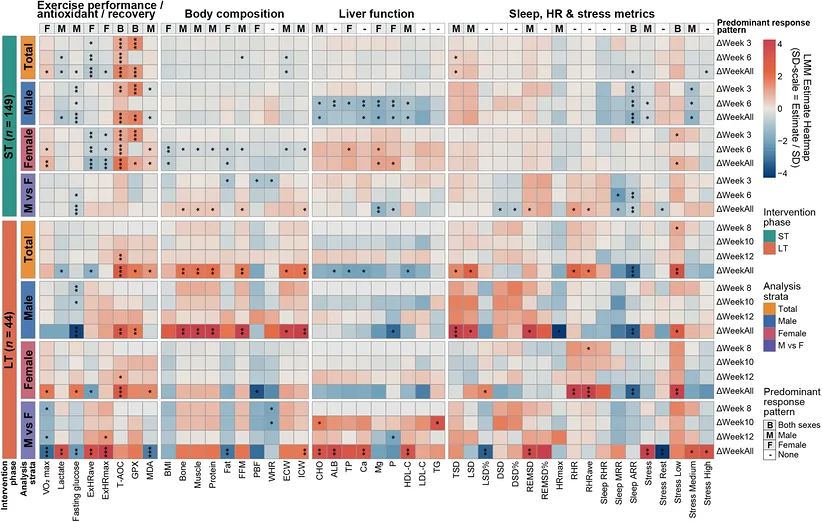
Time‐ and sex‐stratified physiological changes associated with the combined probiotics‐yeast extract formulation. Heatmap of linear mixed‐effects model (LMM) estimates for change from baseline (Δ) in field‐based cardiorespiratory indices, bioelectrical impedance‐derived body‐composition estimates, liver function, and wearable‐derived sleep, heart rate (HR), and stress metrics. Rows: analytical strata (Total, Male, Female, Male vs. Female) within short‐term (ST) or long‐term (LT) phases. Columns: phenotypes grouped by domain. Participant numbers: ST‐Total, *n* = 149 (CPYE, *n* = 99; placebo, *n* = 50); ST‐Male, *n* = 66 (CPYE, *n* = 44; placebo, *n* = 22); ST‐Female, *n* = 83 (CPYE, *n* = 55; placebo, *n* = 28); LT‐Total, *n* = 44 (CPYE, *n* = 31; placebo, *n* = 13); LT‐Male, *n* = 14 (CPYE, *n* = 8; placebo, *n* = 6); LT‐Female, *n* = 30 (CPYE, *n* = 23; placebo, *n* = 7). ST‐Total included 149 participants with post‐baseline data; individual outcome availability varied by measure and visit (Figure S2). Cell color indicates standardized CPYE‐placebo contrast (red = positive; blue = negative). Visit‐specific rows show standardized LMM contrasts for change from baseline at the corresponding visit, whereas the ΔWeekAll row summarizes the overall longitudinal response trajectory across visits. Response‐pattern symbols above the columns indicate whether the predominant nominal pattern was observed in both sexes, in males only, in females only, or in neither sex. For visit‐specific rows, asterisks denote nominal two‐sided Wald *p* values from the LMMs (*p < 0.05, **p < 0.01, ***p < 0.001). For the ΔWeekAll row, asterisks denote *p* values from the permutation‐based trajectory test using the same significance thresholds. No study‐wide multiplicity adjustment was applied across physiological outcomes or visits. The ST‐Total analysis represents the principal randomized comparison; sex‐stratified and LT analyses are exploratory.

During the randomized ST phase, CPYE was associated with time‐dependent differences in field‐based cardiorespiratory indices relative to placebo. Separate permutation‐based longitudinal trajectory tests yielded nominally significant between‐group differences in device‐estimated VO_2_ max (a wearable‐derived, field‐based cardiorespiratory index), ExHRave, and ExHRmax (*p* < 0.05), with directions corresponding to higher device‐estimated VO_2_ max and lower exercise heart‐rate indices in the CPYE group (Figures 2 and 3). These trajectory tests were treated as supportive rather than prospectively confirmatory analyses, and the visit‐specific estimates were not uniformly statistically significant. At Week 6, the estimated CPYE‐placebo difference in change from baseline in device‐estimated VO_2_ max was 0.878 mL/kg/min (95% confidence interval [CI], −0.023 to 1.779; nominal *p* = 0.056). Visit‐specific effect estimates on the original scale, with 95% CIs, are provided in Table S3. The Week 6 post‐exercise lactate contrast was −1.351 mmol/L (95% CI, −2.530 to −0.172; nominal *p* = 0.025) in the primary change‐from‐baseline model but was attenuated in the baseline‐adjusted sensitivity analysis described below. In the exploratory LT phase, initial models suggested lower ExHRave with CPYE; however, after baseline adjustment, the final‐time estimates did not indicate a clear between‐group difference.

**FIGURE 3:**
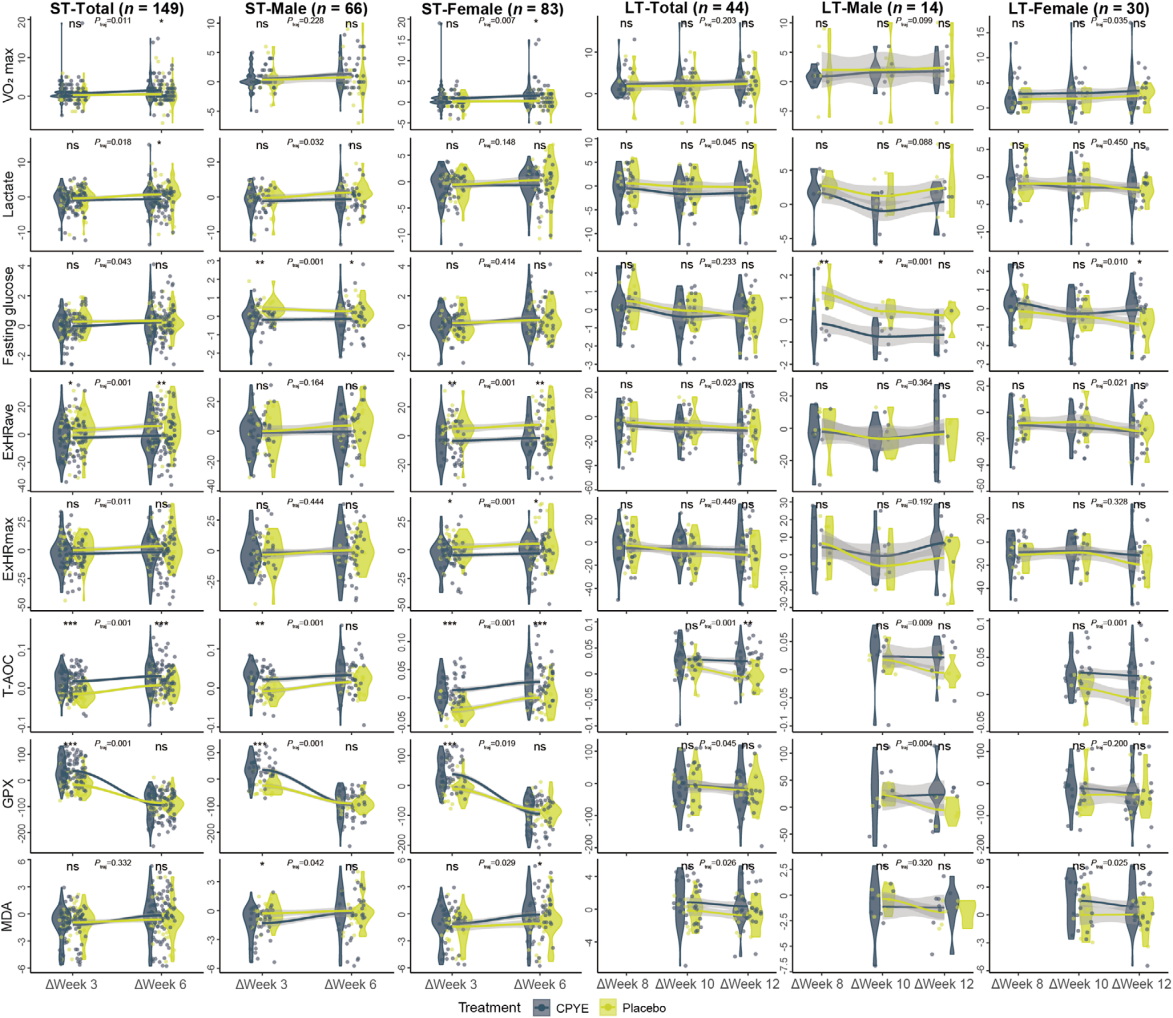
Longitudinal trajectories of selected physiological outcomes. Violin plots of longitudinal changes (Δ from baseline) in device‐estimated VO_2_ max, lactate, fasting glucose, average and maximal exercise heart rate (ExHRave, ExHRmax), total antioxidant capacity (T‐AOC), glutathione peroxidase (GPX) activity, and malondialdehyde (MDA) concentration by treatment (CPYE, blue; placebo, yellow). Panels are stratified by Total, Male, and Female cohorts in short‐term (ST) and long‐term (LT) phases. Dots represent individual values; solid lines indicate group means; shaded bands show 95% confidence intervals. Visit‐specific between‐group comparisons were evaluated using nominal two‐sided Wilcoxon rank‐sum tests: ns, *p* ≥ 0.05; *p < 0.05, **p < 0.01, ***p < 0.001. The upper‐right corner of each panel shows *P*_traj_ from a separate permutation‐based test of the longitudinal trajectory. Visit‐specific and trajectory *p* values are nominal or supportive and were not adjusted across outcomes or visits. All LT and sex‐stratified panels are exploratory and hypothesis‐generating. Additional abbreviations are defined in Table S1.

Concurrently, separate longitudinal trajectory tests indicated that CPYE was associated with shifts in metabolic and redox biomarkers. During the ST phase, lactate and fasting glucose levels were significantly lower in the CPYE group versus placebo (*p* < 0.05), whereas T‐AOC and GPX activity were elevated (Figures 2 and 3). Treatment contrasts varied across visits, with between‐group differences observed at selected time points but not uniformly at both Week 3 and Week 6. Specifically, the T‐AOC and GPX activity were higher in the CPYE group at Week 3 (*p* < 0.05); at Week 6, lactate was lower and T‐AOC remained higher, whereas GPX did not show a clear between‐group difference (Figures 2 and 3; Table S3). Baseline‐adjusted sensitivity analyses for lactate, fasting glucose, and T‐AOC are reported below. Exploratory LT models suggested differences in T‐AOC, GPX, and lactate at select visits.

To evaluate the robustness of exploratory LT treatment, we fitted parsimonious sensitivity models adjusting for the corresponding Week 6 value and sex (Table S2, Panel B). Week 6 values accounted for baseline status at extension entry, and sex was included given its differential distribution between LT entrants and non‐entrants. No additional covariates were added to avoid overfitting in the smaller LT cohort. For device‐estimated VO_2_ max, the original final‐time contrast (CPYE vs. placebo) was 0.525 mL/kg/min (95% CI, −1.938 to 2.989; *p* = 0.673), attenuating to −0.368 mL/kg/min (95% CI, −2.325 to 1.590; *p* = 0.708) after adjustment; both indicated no clear final‐time difference. The initially significant T‐AOC contrast (0.033 µmol/mL; 95% CI, 0.011 to 0.056; *p* = 0.004) attenuated to 0.012 µmol/mL (95% CI, −0.003 to 0.027; *p* = 0.112) following adjustment. Remaining LT outcomes likewise showed imprecise final‐time estimates in both unadjusted and adjusted models, underscoring the exploratory nature of these findings.

Body composition and digital‐physiology metrics showed directionally consistent but smaller‐magnitude associations. During the ST phase, CPYE was associated with slight reductions in fat‐free mass (FFM) and extracellular water (ECW), alongside modest decreases in sleep‐averaged respiratory rate (Sleep ARR) and time in high‐stress states (Figure 2). Exploratory LT analyses suggested modest increases in FFM and skeletal muscle mass, with subtle shifts in wearable‐derived heart‐rate, sleep, and stress metrics, but no substantial changes in total body mass or adiposity. These patterns are presented as hypothesis‐generating and should not be interpreted as confirmatory treatment effects.

Across the randomized ST phase, nominal overall CPYE‐placebo differences were observed for chest discomfort (Q04; nominal *p* = 0.029) and defecatory urgency (Q13; nominal *p* < 0.001), with lower reported frequencies in the CPYE group; the other 12 questionnaire items showed no clear overall differences (all nominal *p* > 0.05; Table S4). Because these item‐level comparisons were not adjusted for multiplicity across the 14 questionnaire outcomes, they should be interpreted cautiously. Item‐level findings in the LT cohort are considered exploratory. The intervention was generally well tolerated: no participant discontinued because of product intolerance, and no adverse events were judged related to the intervention.

To assess the robustness of results to the single ST‐phase withdrawal, we repeated selected ST analyses in the multiple‐imputation intention‐to‐treat cohort (*n* = 150). CPYE‐placebo contrasts closely aligned with those from the 149 participants with observed follow‐up data. For device‐estimated VO_2_ max, observed and imputed estimates were nearly identical at Week 3 (0.401 and 0.403 mL/kg/min) and Week 6 (0.878 and 0.873 mL/kg/min). At Week 6, multiple‐imputation estimates were −1.348 mmol/L for lactate (95% CI, −2.494 to −0.203), −6.501 beats/min for ExHRave (95% CI, −10.961 to −2.041), and 0.024 µmol/mL for T‐AOC (95% CI, 0.013–0.035). The direction of all contrasts was retained under multiple imputation, and the pattern of gastrointestinal questionnaire findings remained unchanged. Thus, the single participant withdrawal did not materially alter the ST effect estimates.

In supportive sensitivity analyses that modeled the original‐scale Week 3 and Week 6 outcomes with adjustment for the corresponding baseline values, estimates from the 149‐participant observed‐data analysis were nearly identical to those from the 150‐participant multiple‐imputation intention‐to‐treat analysis (Table S3). At Week 6, ExHRave remained lower with CPYE (adjusted difference, −4.83 beats/min; 95% CI, −8.64 to −1.02; nominal *p* = 0.013), whereas the lactate contrast attenuated to −0.793 mmol/L (95% CI, −1.653 to 0.068; nominal *p* = 0.071). T‐AOC remained higher at Week 3 (adjusted difference, 0.019 µmol/mL; 95% CI, 0.009 to 0.029; nominal *p* < 0.001), but its Week 6 contrast attenuated to 0.006 µmol/mL (95% CI, −0.004 to 0.016; nominal *p* = 0.250). Device‐estimated VO_2_ max at Week 6 (adjusted difference, 0.771 mL/kg/min; 95% CI, −0.092 to 1.634; nominal *p* = 0.080) and fasting glucose at both follow‐up visits showed no clear between‐group differences after adjustment.

Collectively, the randomized ST analyses revealed CPYE‐placebo differences in field‐based cardiorespiratory, metabolic, and redox indices, together with nominal overall differences in two of the 14 gastrointestinal questionnaire items, without a consistent questionnaire‐wide pattern. The magnitude and direction of these contrasts varied across outcomes, motivating further exploration stratified by intervention phase and sex. LT findings are reported separately as a post‐randomization selected cohort; sensitivity analyses indicated that most final‐time estimates were imprecise and that nominal significance observed in unadjusted models was attenuated by covariate adjustment.

### 2.3 Exploratory Sex‐ and Phase‐Stratified Physiological Response Patterns

Given heterogeneity in baseline cardiorespiratory fitness and body composition, we explored whether pre‐intervention phenotypes could stratify intervention responses. Unsupervised hierarchical clustering of *z*‐scored baseline measures (device‐estimated VO_2_ max, heart rate indices, adiposity, and FFM) identified a two‐cluster solution with maximal average silhouette width (Figure S3a). Cluster assignment showed 91.28% concordance with biological sex (136/149 participants; Figure S3b), indicating that cluster membership largely reflected sex in this cohort.

Accordingly, we re‐fitted LMMs within sex‐stratified subgroups across ST and LT phases (Figures 2 and 3), yielding four exploratory response patterns:

(1) ST males: Metabolic and redox‐associated shifts

In male athletes, CPYE was associated with reduced lactate and fasting glucose (*p* < 0.05 vs. placebo) without a significant change in device‐estimated VO_2_ max. Concurrently, T‐AOC and GPX activity increased, while MDA decreased (*p* < 0.05). Wearable‐derived heart‐rate, sleep, and stress metrics showed directional differences that are presented as exploratory and should not be interpreted as evidence of improved sleep quality or autonomic function.

(2) ST females: Concurrent shifts in field‐based cardiorespiratory indices and body composition

In female athletes, CPYE was associated with increased device‐estimated VO_2_ max, T‐AOC, and GPX activity, alongside reductions in both ExHRave and ExHRmax (*p* < 0.05). Concurrent reductions in body mass, adiposity, and total body water occurred together with increases in serum total protein, magnesium, and phosphorus. Time spent in low‐stress states also increased significantly (*p* < 0.05). These patterns represent concurrent associations across field‐based cardiorespiratory indices, bioelectrical impedance‐derived body‐composition estimates, and wearable‐derived stress metrics within the habitual training context.

(3) LT males: Exploratory associations with lean‐tissue estimates and redox biomarkers

In the exploratory LT male subset, CPYE was associated with higher FFM, skeletal muscle mass, bone mass, protein mass, ECW, intracellular water (ICW), T‐AOC, and GPX activity, alongside lower fasting glucose, and Sleep ARR (*p* < 0.05). These findings suggest concurrent associations between bioelectrical impedance‐derived lean‐tissue estimates and redox‐related biomarkers without a corresponding increase in estimated fat mass.

(4) LT females: Mixed associations across adiposity, cardiorespiratory, and cardiometabolic measures

In the LT female subset, CPYE was associated with lower PBF and higher device‐estimated VO_2_ max, alongside lower ExHRave, elevated T‐AOC, and more time in low‐stress states (*p* < 0.05). However, fasting glucose and MDA showed modest increases, and both resting heart rate (RHR) and averaged RHR (RHRave) rose slightly, while Sleep ARR decreased. Within the intensified‐training context, these findings indicate mixed associations across adiposity estimates, field‐based cardiorespiratory indices, cardiometabolic measures, and redox biomarkers. The concurrent reduction in Sleep ARR and extension of low‐stress states should be interpreted cautiously, as these wearable‐derived metrics do not directly establish sleep quality or autonomic adaptation.

Overall, these exploratory analyses revealed heterogeneous patterns across sex and phase rather than uniform responses. ST males primarily showed metabolic and redox‐associated shifts, whereas ST females showed concurrent changes in field‐based cardiorespiratory indices and body‐composition estimates. Within the selected LT cohort, male and female strata exhibited different patterns in lean‐tissue and adiposity estimates, respectively. Because baseline physiological clusters showed 91.28% concordance with sex, we also explored baseline physiology as a potential source of heterogeneity. Cluster‐stratified LMMs yielded largely concordant effect directions (Figure S3c), but the substantial overlap between cluster membership and biological sex precludes disentangling their independent contributions. All sex‐stratified and cluster‐stratified findings are presented as exploratory and hypothesis‐generating.

### 2.4 Gut Multi‐Omics Variation by Intervention Phase and Sex

To examine whether time‐ and sex‐associated physiological patterns occurred with gut ecosystem variation, we integrated multi‐omics profiles spanning bacterial, fungal, and phage communities, microbial functional pathways, and fecal metabolites.

Global permutational multivariate analysis of variance (PERMANOVA) based on Aitchison distances across all 577 samples showed that intervention phase (ST vs. LT), treatment (CPYE vs. placebo), and sex were each associated with variation across multiple omics layers (summed pseudo‐*F* = 28.49, 25.18, and 18.49, respectively; Table S5). These multivariable associations reflect descriptive patterns rather than direct host‐microbiome coupling (Figure S4). Given the comparatively large phase‐associated statistic, we stratified subsequent analyses by phase. Within both ST and LT phases, treatment and sex remained associated with variation. Notably, in the LT phase, sex yielded a larger summed pseudo‐*F* than treatment (32.53 vs. 29.06; *p* < 0.05; Figure S4b), suggesting that within the selected LT cohort, sex was associated with variation in microbial and metabolic profiles. This descriptive pattern supports explicit consideration of sex in microbiome‐targeted trials.

To dissect these interactions, we performed sex‐ and phase‐stratified PERMANOVA (Figure S4a,b). In ST females, treatment yielded the largest summed pseudo‐*F* (12.84), indicating early treatment‐associated separation in these exploratory analyses. In ST males, however, host serum lipid measures, specifically total cholesterol (16.40) and high‐density lipoprotein cholesterol (15.66), showed larger summed pseudo‐*F* values than treatment (15.29), suggesting that lipid profiles were also associated with early microbial variation in men. Distinct patterns emerged in the LT phase: in LT males, treatment yielded the largest summed pseudo‐*F* (32.01), whereas in LT females, host cardiorespiratory and skeletal phenotypes (device‐estimated VO_2_ max: 30.02; bone mass: 26.20) showed comparatively large associations with microbial functional and metabolic composition, with a smaller but statistically significant treatment‐associated statistic (23.92; Figure S4b). These exploratory patterns suggest sex‐ and phase‐associated heterogeneity but do not establish temporal coupling between microbiome and physiological responses.

To leverage the longitudinal data structure, we applied compositional tensor factorization (CTF) across all omics layers (Figure 4). Unstratified CTF embeddings showed separation between CPYE and placebo trajectories, particularly for bacterial and functional profiles (Figure 4a). PERMANOVA in CTF space identified treatment‐associated separation in the ST phase for bacteria (pseudo‐*F* = 3.76) and microbial functions (pseudo‐*F* = 16.29; both *p* < 0.05; Figure 4b). In the selected LT cohort, treatment‐associated separation was observed across bacteria (pseudo‐*F* = 14.68), fungi (pseudo‐*F* = 12.51), phages (pseudo‐*F* = 6.47), and functions (pseudo‐*F* = 15.16; all *p* < 0.01). Between‐group metabolite separation was not detected in the unstratified LT analysis but was observed in specific sex‐stratified LT comparisons.

**FIGURE 4:**
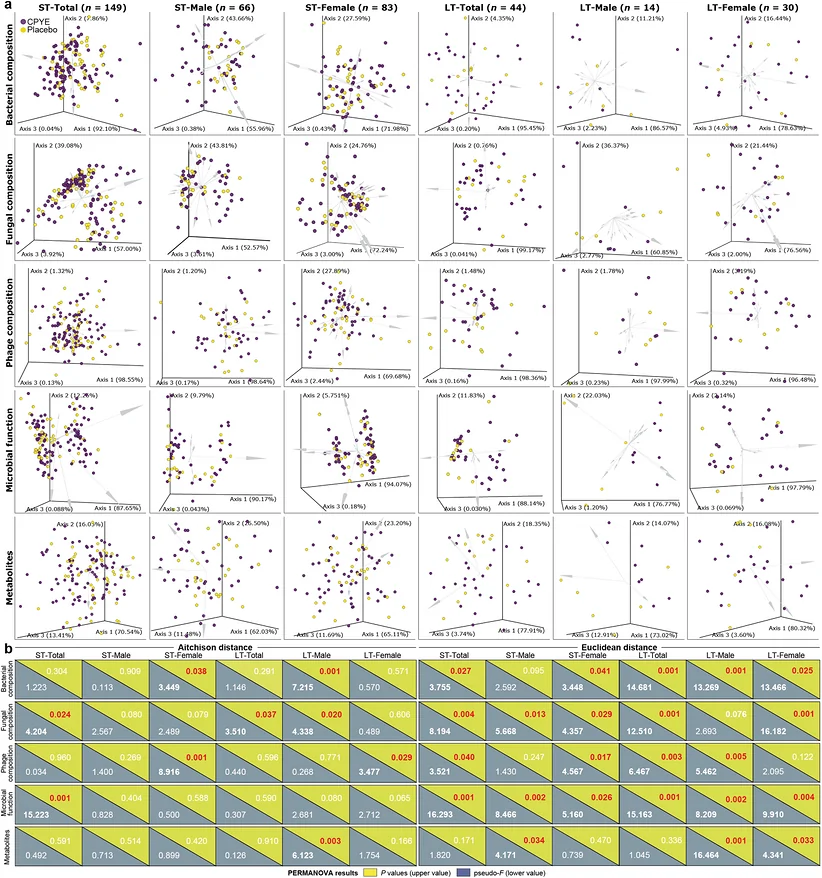
Combined probiotics‐yeast extract formulation‐associated variation in the gut multi‐omics landscape across time and sex strata. (a) Compositional tensor factorization (CTF) ordinations of bacterial, fungal, phage, microbial functional, and fecal metabolite profiles during short‐term (ST) and long‐term (LT) phases in total and sex‐stratified cohorts. Each dot represents one sample colored by treatment (combined probiotics‐yeast extract [CPYE], purple; placebo, yellow). Axes correspond to the first three CTF components (percentage of variation in parentheses). Ordinations provide descriptive visualization of treatment‐group and temporal patterns; statistical comparisons are presented in panel (b). (b) PERMANOVA comparisons of CPYE and placebo profiles in CTF space for each omics layer and phase‐by‐sex stratum. Lower triangle: pseudo‐*F* statistic; upper triangle: nominal permutation *p* value (999 permutations). *p* < 0.05 shown in red. Left: Aitchison distance; right: Euclidean distance. Participant numbers are shown. A total of 577 unique fecal samples contributed to the multi‐omics dataset: 447 from the ST phase (Weeks 0–6), 262 from the LT extension (Weeks 6–12), with 132 ST phase samples included in both analytical windows.

Sex‐stratified CTF analyses suggested heterogeneous temporal patterns (Figure 4a). In females, significant CPYE‐placebo separation emerged as early as the ST phase across bacteria (pseudo‐*F* = 3.45), fungi (pseudo‐*F* = 4.36), phages (pseudo‐*F* = 4.58), and functions (pseudo‐*F* = 5.16; all *p* < 0.05), with metabolite separation appearing only in the LT phase (pseudo‐*F* = 4.34, *p* < 0.05). In males, the early response was more restricted: significant ST‐phase separation was limited to fungi (pseudo‐*F* = 5.67), functions (pseudo‐*F* = 8.47), and metabolites (pseudo‐*F* = 4.17; *p* < 0.05), with bacterial and metabolic profiles showing the strongest LT divergence (bacteria pseudo‐*F* = 13.27, metabolites pseudo‐*F* = 16.46; *p* < 0.05). Thus, these exploratory embeddings suggested earlier separation of microbial community structure and function in females, whereas males showed more pronounced separation of bacterial and metabolic profiles in the selected LT cohort.

Finally, α‐diversity metrics (Shannon and Simpson indices, species richness) showed no consistent differences across treatment groups, time points, or sex strata (Figure S5). Thus, observed compositional differences were not accompanied by systematic shifts in these diversity metrics; this finding does not establish ecological benefit or stability.

### 2.5 CPYE‐Associated Shifts in Gut Microbial Taxa, Functions, and Metabolites by Phase and Sex

To identify feature‐level associations with the observed physiological and community‐level patterns, we performed differential abundance analysis using Microbiome Multivariable Association with Linear Models (MaAsLin2), modeling treatment, time, age, and BMI as fixed effects and participant as a random intercept. Analyses were stratified by intervention phase (ST and LT) and biological sex. Using an exploratory screening threshold of Benjamini–Hochberg‐adjusted *q* < 0.25, we retained 823 candidate features: 59 bacterial taxa, 11 fungal taxa, one phage taxon, 422 microbial functional pathways, and 330 metabolites (Figure 5).

**FIGURE 5:**
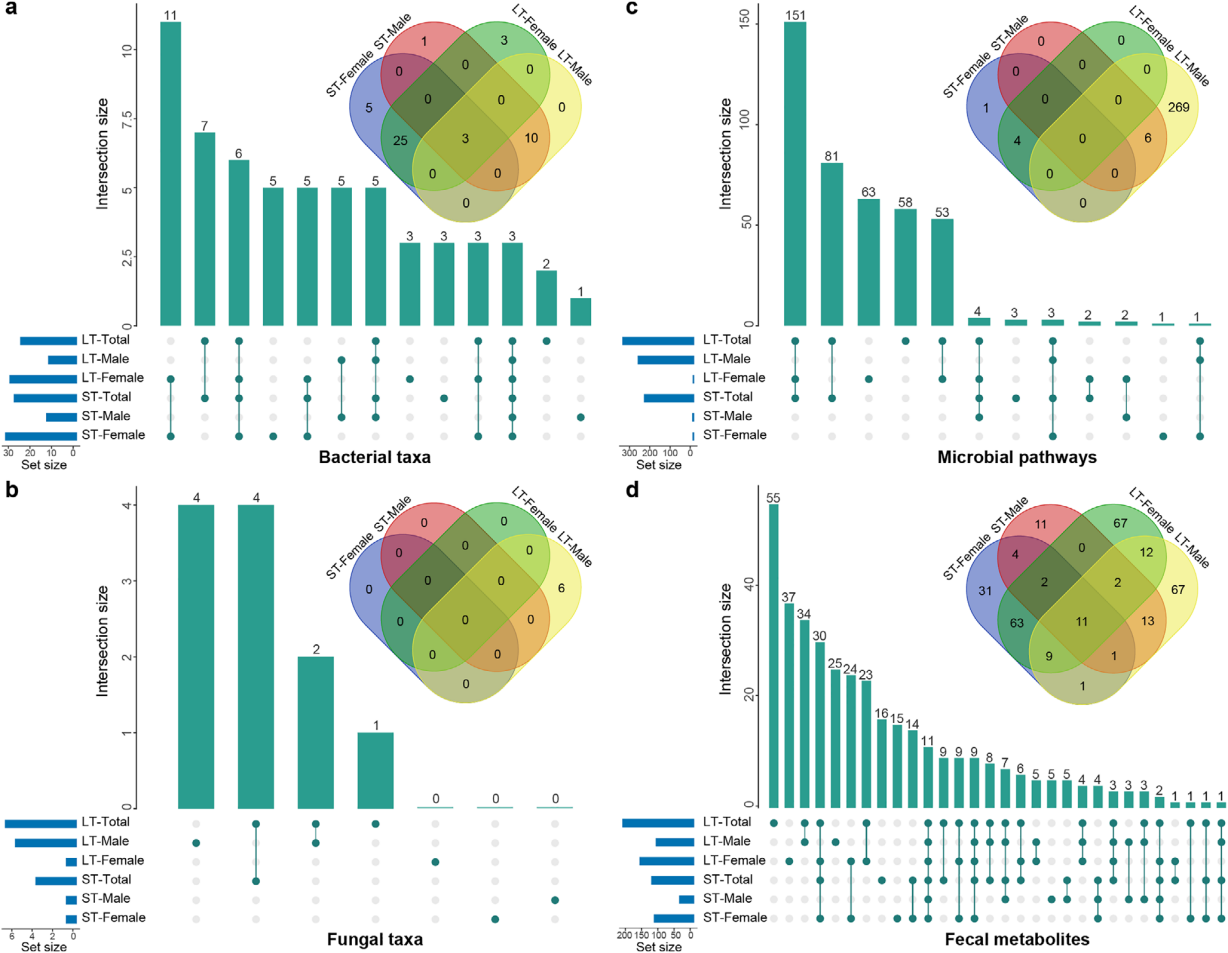
Combined probiotics‐yeast extract formulation‐associated multi‐omics features are largely non‐overlapping across sex and time. UpSet plots and sex‐stratified Venn diagrams summarize candidate‐associated features identified by MaAsLin2 (*q* < 0.25) in short‐term (ST) and long‐term (LT) phases. ST analyses: 149 participants (66 males, 83 females); LT analyses: 44 participants (14 males, 30 females). Features were prefiltered at ≥ 30% prevalence and analyzed using MaAsLin2 with treatment, time, age, and body mass index as fixed effects and participant as a random effect. Benjamini–Hochberg adjustment was performed within each analysis; *q* < 0.25 was used as the exploratory screening threshold. Panels: (a) bacterial taxa; (b) fungal taxa; (c) microbial pathways; (d) fecal metabolites. In UpSet plots, horizontal bars indicate total candidate features per stratum; vertical bars represent intersection sizes. Venn diagrams show limited cross‐sex overlap, consistent with largely stratum‐specific exploratory patterns.

Retained features differed across phase and sex strata, with minimal overlap. During the ST phase, candidate features were sparse and predominantly sex‐specific. At the bacterial level, male and female athletes exhibited largely non‐overlapping sets of differentially abundant taxa (Figure 5a). Fungal shifts were detected exclusively in LT males, whereas a greater number of candidate metabolite differences were retained in ST females (Figure 5b,d). Microbial functional differences were present but limited in number, with no shared features between sexes (Figure 5c).

In the LT phase, more candidate features were retained, particularly among microbial functions and metabolites, yet patterns remained largely sex‐specific. The majority of LT‐associated pathways and metabolites were altered in only one sex, with negligible cross‐sex overlap (Figure 5c,d). Bacterial signatures increased modestly in number compared with ST but continued to be dominated by sex‐specific taxa, with only a small shared core (Figure 5a). Notably, a single phage taxon (family *Autographiviridae*) was identified as decreased exclusively in LT females.

Across all phases and strata, only a minimal core of features showed consistent association with CPYE. This core included taxa such as *Lacticaseibacillus*, *Bifidobacterium animalis* subsp. *lactis*, and *Lacticaseibacillus paracasei* (the species containing the administered strain Zhang), alongside a limited set of metabolites. The restricted overlap suggests that CPYE‐associated microbial and metabolic features were highly context‐dependent across intervention duration and sex. All feature‐level associations are exploratory and hypothesis‐generating and should not be interpreted as evidence of a defined ecosystem‐remodeling mechanism.

### 2.6 CPYE‐Associated Differences in Gut Multi‐Omics Network Topology and Simulated Robustness

Beyond compositional shifts, we compared the topology of CPYE‐ and placebo‐derived gut multi‐omics co‐occurrence networks. The 823 candidate‐associated microbial and metabolic features served as nodes; edges were defined by Spearman correlations of centered log‐ratio‐transformed abundances. Networks were generated separately by intervention phase, treatment group, and sex stratum. Because they were constructed from a pre‐screened feature set (*q* < 0.25), these analyses are interpreted as descriptive selected‐feature networks.

Network organization was summarized by edge count, density, average path length (APL), largest connected component (LCC) ratio, and degree heterogeneity. Robustness to targeted (*R*target) and random (*R*random) node removal was evaluated by simulation; higher values indicate greater robustness within the specified network model rather than directly measured ecological stability in vivo.

In the full cohort, CPYE‐derived networks showed higher connectivity and simulated robustness metrics than placebo‐derived networks in both phases (Table 2). During the ST phase, CPYE and placebo networks had 18 813 vs. 12 779 edges, densities of 0.06 vs. 0.04, APL values of 2.58 vs. 3.80, and LCC ratios of 0.93 vs. 0.84, respectively. *R*target was 0.37 vs. 0.26 and *R*random was 0.93 vs. 0.84. During the LT phase, corresponding values were 24 962 vs. 15 597 edges, densities of 0.07 vs. 0.05, APL values of 2.43 vs. 2.97, and LCC ratios of 0.97 vs. 0.90; *R*target was 0.41 vs. 0.32 and *R*random was 0.96 vs. 0.89. Degree heterogeneity was lower in CPYE‐derived networks (ST: 0.86 vs. 1.28; LT: 0.83 vs. 1.07), indicating less hub‐dominated topology within these inferred networks.

**TABLE 2:** Topological properties of gut multi‐omics co‐occurrence networks by intervention group, sex, and phase.

Network metric	CPYE						Placebo					
	Short‐term			Long‐term			Short‐term			Long‐term		
	Total	Male	Female	Total	Male	Female	Total	Male	Female	Total	Male	Female
Nodes	823	823	823	823	823	823	823	823	823	823	823	823
Edges	18813	2414	9661	24962	4741	31573	12779	2601	1506	15597	4250	3868
Density	0.056	0.007	0.029	0.074	0.014	0.093	0.038	0.008	0.004	0.046	0.013	0.011
Heterogeneity	0.856	0.959	0.983	0.832	0.941	0.736	1.275	0.849	0.893	1.065	1.324	0.977
*R*target	0.372	0.083	0.288	0.413	0.176	0.439	0.255	0.031	0.037	0.321	0.087	0.146
*R*random	0.925	0.537	0.878	0.964	0.722	0.972	0.835	0.314	0.361	0.893	0.546	0.703
Average path length	2.576	6.385	3.264	2.433	3.916	2.232	3.797	5.213	5.693	2.972	5.964	5.236
Largest connected component ratio	0.934	0.542	0.887	0.973	0.729	0.982	0.843	0.317	0.365	0.902	0.552	0.710

*Note*: Networks were constructed from 823 combined probiotics‐yeast extract (CPYE)‐associated microbial and metabolite features (FDR *q* < 0.25 screening) using Spearman correlations on centered log‐ratio‐transformed abundances. Short‐term = Weeks 0–6; long‐term = Weeks 6–12. *R*target (robustness to targeted removal) and *R*random (robustness to random removal) describe simulated robustness to node removal under the specified network model (higher = greater simulated robustness). Density = 2 × edges / [nodes × (nodes – 1)] for undirected networks. Heterogeneity = coefficient of variation of node degree (SD/mean); higher values indicate more hub‐dominated topology. These metrics describe inferred network topology and should not be interpreted as direct measures of in vivo ecological stability, functional integration, or resistance to physiological perturbation.

Sex‐stratified networks showed heterogeneous topology, with the largest numerical contrasts in the small LT female subgroup. CPYE‐ and placebo‐derived LT female networks had 31 573 vs. 3868 edges, densities of 0.09 vs. 0.01, APL values of 2.23 vs. 5.24, and LCC ratios of 0.98 vs. 0.71, respectively. *R*target was 0.44 vs. 0.15 and *R*random was 0.97 vs. 0.70. In LT males, corresponding networks had 4741 vs. 4250 edges, APL values of 3.92 vs. 5.96, and LCC ratios of 0.73 vs. 0.55; *R*target was 0.18 vs. 0.09 and *R*random was 0.72 vs. 0.55.

Differences were also observed during the ST phase. In females, CPYE‐ and placebo‐derived networks had 9661 vs. 1506 edges, densities of 0.030 vs. 0.004, APL values of 3.26 vs. 5.69, and LCC ratios of 0.89 vs. 0.37; *R*target was 0.29 vs. 0.04 and *R*random was 0.88 vs. 0.36. In males, the CPYE‐derived network had slightly fewer edges than the placebo network (2414 vs. 2601) but a larger LCC ratio (0.54 vs. 0.32), higher *R*target (0.08 vs. 0.03), and higher *R*random (0.54 vs. 0.31). Thus, observed topology was not characterized by a uniform increase in edge count across all strata.

Collectively, CPYE‐ and placebo‐derived networks differed in connectivity, path structure, component size, hub dominance, and simulated node‐removal behavior, with the largest contrasts observed in the selected LT female subgroup. These results identify sex‐ and phase‐associated systems‐level patterns for further study but do not establish in vivo ecological stability, functional resilience, or a mechanistic link between network topology and host physiology.

### 2.7 Exploratory Indirect‐Effect Analyses Linking CPYE, Gut Features, and Physiological Outcomes

To examine statistical pathways linking CPYE exposure, candidate gut features, and host outcomes, we applied hierarchical linear mediation models to high‐influence nodes from the multi‐omics networks (degree ≥ 3; normalized edge strength heterogeneity ≥ 0.8). Models were fitted within each sex‐phase stratum, adjusting for age, BMI, and ethnicity, with participant modeled as a random intercept (Figures 6 and 7; Table S6). Although the statistical procedure yields values labeled as average causal mediation effects, we report these as exploratory indirect‐effect estimates; they describe statistical associations and do not establish causal mediation or directed biological pathways.

**FIGURE 6:**
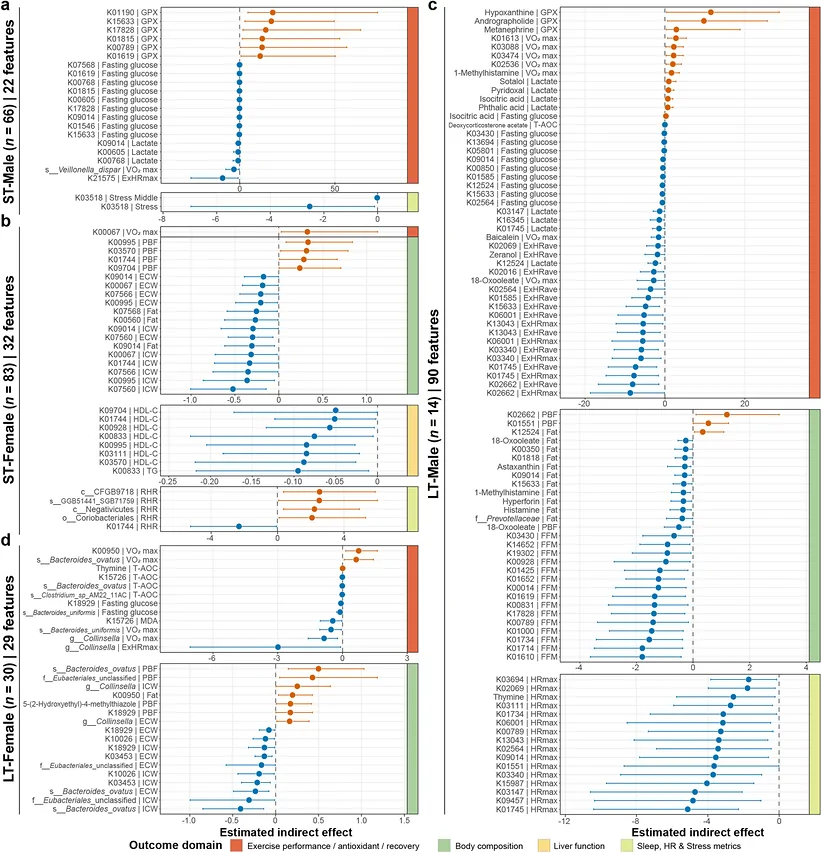
Exploratory indirect‐effect estimates linking microbial and metabolic features to physiological outcomes. Forest plots show model‐based indirect‐effect estimates (statistically labeled as average causal mediation effects) for high‐influence network nodes (degree ≥ 3; normalized edge‐strength heterogeneity ≥ 0.8). Rows: candidate features (taxa, pathways, metabolites) grouped by host outcome domain (red = field‐based cardiorespiratory, antioxidant, and recovery‐related outcomes; green = bioelectrical impedance‐derived body‐composition estimates; yellow = serum biochemical measures; pale green = wearable‐derived sleep and stress metrics). Dots show point estimates; horizontal whiskers show 95% confidence intervals. Orange and blue points indicate positive and negative estimates, respectively; dashed vertical lines denote zero. Panels (a,b) show short‐term (ST) male and female strata, and panels (c,d) show long‐term (LT) male and female strata, respectively. Models were adjusted for age, body mass index, and ethnicity, with participant as a random effect. Analytic participant and observation counts varied by omics feature and outcome availability (Figure S2). Within each outcome‐domain block, feature‐outcome pairs are ordered by absolute magnitude of the indirect‐effect estimate. Because candidate features were selected and evaluated within the same dataset, all sex‐stratified and LT indirect‐effect results are exploratory and hypothesis‐generating. Additional abbreviations are defined in Table S1.

**FIGURE 7:**
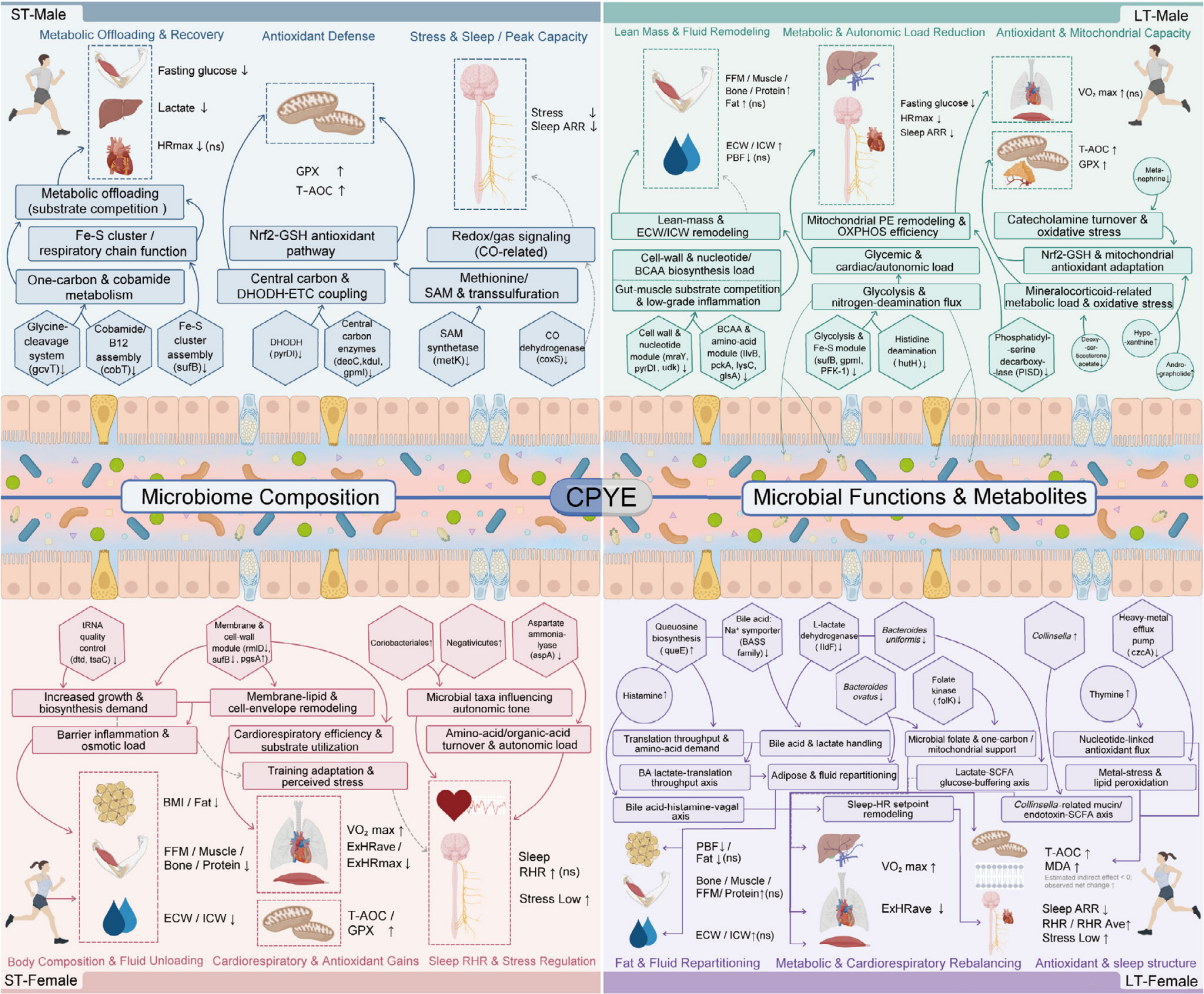
Hypothesis‐generating model of time‐ and sex‐associated candidate pathways linking combined probiotics‐yeast extract exposure to host physiology. Schematic integrating candidate associations from physiological, multi‐omics network, and exploratory indirect‐effect analyses. Male (top) and female (bottom) response patterns are shown for short‐term (ST; left) and long‐term (LT; right) phases. Representative candidate microbial‐metabolic modules include one‐carbon/B_12_/Fe‐S metabolism, membrane‐related functions, bile acid and lactate metabolism, and folate‐ and histamine‐related functions. Solid and dashed arrows summarize model‐derived associations and proposed hypotheses, respectively; neither denotes an experimentally validated causal pathway. Upward and downward arrows adjacent to measured outcomes indicate the observed direction of corresponding estimates; ns denotes non‐significant comparisons. All sex‐stratified and LT patterns are exploratory. Additional abbreviations are defined in Table S1.

### 2.8 ST Males: Candidate Pathways Involving Microbial One‐Carbon and Redox Metabolism

In ST males, CPYE was associated with lower lactate and fasting glucose, alongside higher T‐AOC and GPX activity and lower wearable‐derived stress measures (Figures 6a and 7). Negative indirect effects for lactate and fasting glucose were associated with reduced relative abundance of microbial functions including aminomethyltransferase (K00605, *gcvT*), nicotinate‐nucleotide‐dimethylbenzimidazole phosphoribosyltransferase (K00768, *cobT*), and Fe‐S cluster assembly protein SufB (K09014, *sufB*). The annotated roles of these functions place microbial one‐carbon, cobamide/B_12_, and Fe‐S assembly pathways among candidate processes associated with early metabolic pattern; however, these estimates do not demonstrate altered microbial substrate use or host metabolic flux [22, 23].

Indirect‐effect estimates for antioxidant outcomes involved reduced abundance of S‐adenosylmethionine synthetase (K00789, *metK*), dihydroorotate dehydrogenase, NAD^+^ (K17828, *pyrDI*), deoxyribose‐phosphate aldolase (K01619, *deoC*), 4‐deoxy‐L‐threo‐5‐hexosulose‐uronate ketol‐isomerase (K01815, *kduI*), and 2,3‐bisphosphoglycerate‐independent phosphoglycerate mutase (K15633, *gpmI*), which showed positive indirect effects GPX. These statistical associations connect methionine/S‐adenosylmethionine, pyrimidine, and central‐carbon functions with redox‐related outcomes, generating hypotheses for targeted studies of glutathione synthesis and mitochondrial metabolism [22, 23, 24]. Aerobic carbon monoxide dehydrogenase (K03518, *coxS*) also showed a negative indirect effect for stress scores, nominating an additional gas‐ and redox‐related candidate [25, 26, 27].

### 2.9 ST Females: Candidate Microbial Biosynthetic Functions Linked to Physiological Outcomes

In ST females, CPYE was associated with concurrent shifts in device‐estimated VO_2_ max, exercise heart‐rate indices, redox‐related biomarkers, and bioelectrical impedance‐derived body‐composition estimates, including BMI, total fat mass, FFM, skeletal muscle mass, bone mass, protein mass, total body water, PBF, and waist‐hip ratio (Figures 6b and 7). Indirect‐effect models identified reduced relative abundance of dTDP‐4‐dehydrorhamnose reductase (K00067, *rmlD*), Fe‐S cluster assembly protein SufB (K09014, *sufB*), *D*‐aminoacyl‐tRNA deacylase (K07560, *dtd*), and *L*‐threonylcarbamoyladenylate synthase (K07566, *tsaC*), but an increased relative abundance of CDP‐diacylglycerol‐glycerol‐3‐phosphate 3‐phosphatidyltransferase (K00995, *pgsA*). Except for *pgsA*, these features showed negative indirect effects for FFM, skeletal muscle mass, bone mass, protein mass, and ECW/ICW.

These functions annotate cell‐wall biosynthesis, phospholipid metabolism, Fe‐S assembly, and tRNA quality control, providing biological context for candidate associations with body‐composition and fluid‐balance estimates [28, 29, 30]. Separately, *rmlD* showed a positive indirect effect for device‐estimated VO_2_ max, while aspartate ammonia‐lyase (K01744, *aspA*) showed a negative indirect effect for sleep‐related RHR. Taxonomic features from Negativicutes and Coriobacteriales showed opposing positive indirect estimates [29, 31, 32, 33]. These statistical links prioritize microbial biosynthetic and membrane‐related candidates without establishing tissue remodeling, autonomic regulation, or a defined physiological mechanism.

### 2.10 LT Males: Exploratory Associations with Amino Acid, Nucleotide, and Redox‐Related Pathways

In the selected LT male subgroup, CPYE was associated with higher FFM, skeletal muscle mass, bone mass, and protein mass; differences in ECW/ICW estimates; and similar total fat mass and PBF relative to placebo. Lower fasting glucose, HRmax, and Sleep ARR, alongside higher T‐AOC and GPX activity, were also observed (Figures 6c and 7). Because this subgroup was selected after randomization and was small, all indirect‐effect results are hypothesis‐generating. Negative indirect effects for FFM, skeletal muscle mass, bone mass, protein mass, and ECW/ICW corresponded to reduced relative abundance of microbial functions related to peptidoglycan, nucleotide, branched‐chain amino acid, and energy metabolism: phospho‐*N*‐acetylmuramoyl‐pentapeptide transferase (K01000, *mraY*), dihydroorotate dehydrogenase (K17828, *pyrDI*), uridine kinase (K00876, *udk*), acetolactate synthase (K01652, *ilvB*), phosphoenolpyruvate carboxykinase (K01610, *pckA*), aspartate kinase (K00928, *lysC*), and glutaminase (K01425, *glsA*). These associations nominate amino acid‐ and nucleotide‐related pathways for validation rather than demonstrating reduced host‐microbe substrate competition or lean‐tissue synthesis [34, 35].

For metabolic and heart‐rate outcomes, reduced relative abundance of Fe‐S cluster assembly protein SufB (K09014, *sufB*), 2,3‐bisphosphoglycerate‐independent phosphoglycerate mutase (K15633, *gpmI*), phosphofructokinase‐1 (K00850, *PFK‐1*), and histidine ammonia‐lyase (K01745, *hutH*) showed negative indirect effects for fasting glucose, ExHRmax, and sleep‐related heart‐rate markers. These functions provide candidate links to glycolysis, deamination, and amine metabolism [25, 36], but their influence on host glycemia or wearable‐derived physiology remains to be tested.

For antioxidant‐related outcomes, deoxycorticosterone acetate showed reduced relative abundance in the CPYE group and a negative indirect effect for T‐AOC. Positive indirect effects for GPX were associated with increased andrographolide and hypoxanthine, whereas reduced metanephrine also showed a positive indirect effect. Prior literature links andrographolide to Nrf2‐related redox biology and hypoxanthine to purine turnover [22, 37, 38, 39, 40, 41], providing context for these candidates without demonstrating pathway activation. Phosphatidylserine decarboxylase (K01613, *PISD*)‐linked functions additionally showed a positive indirect effect for device‐estimated VO_2_ max, nominating a membrane‐related candidate pathway for experimental evaluation [42, 43, 44].

### 2.11 LT Females: Exploratory Associations with Bile Acid, Lactate, and Folate‐Related Pathways

In the selected LT female subgroup, CPYE was associated with lower PBF, higher device‐estimated VO_2_ max, lower ExHRave, higher T‐AOC, and more time in low‐stress states (Figures 6d and 7). Fasting glucose and MDA increased moderately, RHR and RHRave rose slightly, and Sleep ARR decreased, indicating mixed directions across the measured metabolic, redox‐related, and wearable‐derived outcomes. These subgroup findings and corresponding indirect‐effect estimates remain exploratory.

For body‐composition outcomes, *B. ovatus* and *L*‐lactate dehydrogenase complex protein (K18929, *lldF*) showed positive indirect effects for PBF [35, 45, 46]. Bile acid:Na^+^ symporter (K03453, BASS family), *lldF*, and tRNA‐queuosine biosynthesis enzyme (K10026, *queE*) showed negative indirect effects for bone mass, FFM, skeletal muscle mass, protein mass, and ECW/ICW; BASS and *lldF* were reduced under CPYE. Together, these results nominate bile acid‐, lactate‐, and amino acid‐related features as candidate correlates of the body‐composition pattern [47, 48].

For field‐based cardiorespiratory and redox‐related outcomes, *B. ovatus* and folate kinase (K00950, *folK*) showed positive indirect effects for device‐estimated VO_2_ max. Given the documented folate‐producing capacity of *B. ovatus* and the established role of *folK* in microbial folate biosynthesis, these findings nominate folate‐related candidates for further investigation [49, 50]. *Collinsella* abundance increased under CPYE and showed negative indirect effects for device‐estimated VO_2_ max, illustrating that candidate features did not uniformly align with the overall physiological pattern [30, 36]. Although fasting glucose was higher at the group level, *lldF* and *B. uniformis* were reduced under CPYE and showed negative indirect effects for fasting glucose, nominating additional lactate‐related associations [8, 30, 51].

For oxidative stress, reduced relative abundance of the heavy‐metal efflux pump CzcA (K15726, *czcA*) showed a negative indirect effect for MDA, while increased thymine showed a positive indirect effect for T‐AOC. Previous evidence linking *czcCBA*‐mediated metal homeostasis to bacterial responses to metal‐induced oxidative stress provides biological context for the *czcA* finding [52], whereas the established reactivity of thymine with hydroxyl radicals offers only tentative chemical context for the thymine–T‐AOC association [53]. Resting heart rate and Sleep ARR showed indirect associations involving BASS‐family transporters, *queE*, histamine, and *folK*, nominating bile acid‐, histamine‐, and nucleotide‐related candidates [54, 55]. These wearable‐derived measures do not directly establish sleep quality or autonomic adaptation.

Across sex‐phase strata, models linked distinct microbial features to concurrent physiological outcomes, prioritizing candidates for targeted validation. Features were selected via false discovery rate screening and network filters; indirect‐effect models were then fitted to this subset, with outputs reported at nominal thresholds. Because selection was observational, mediator‐outcome confounding and temporal ordering remain unverified, and no experimental perturbation was performed. These exploratory estimates therefore support the hypothesis‐generating framework in Figure 7 without establishing causal pathways; effect directions reflect the screening context, and LT findings warrant additional caution due to post‐randomization selection and limited subgroup sizes.

## 3 Discussion

In this two‐stage trial, we evaluated whether a food‐grade microbiome‐targeted intervention was associated with physiological and gut ecosystem responses in 150 collegiate endurance athletes during a 6‐week randomized ST phase and a post‐randomization selected LT extension through Week 12. The randomized ST phase provided the principal intervention comparison, whereas the LT extension, sex‐stratified analyses, cluster‐stratified analyses, inferred networks, and indirect‐effect models were exploratory and hypothesis‐generating. Rather than indicating a uniform response, the findings suggested patterns that varied with intervention phase, sex, and baseline physiological profile and were accompanied by distinct microbial and metabolic signals.

Multiple‐imputation intention‐to‐treat estimates closely aligned with those from participants with observed follow‐up data, indicating that the single ST withdrawal was unlikely to explain the overall pattern of treatment contrasts. Delta‐adjusted sensitivity analyses similarly preserved the direction of all eight selected physiological contrasts across specified missing‐outcome scenarios. These results support the robustness of ST effect estimates to alternative handling of the single missing record, although they cannot address unexamined missing‐data mechanisms.

Exploratory sex‐stratified analyses revealed heterogeneous time‐ and sex‐resolved physiological patterns. During the ST phase, male athletes showed lower post‐exercise lactate and fasting glucose together with higher GPX activity and T‐AOC relative to placebo, a pattern consistent with reduced exercise‐related metabolic load and elevated antioxidant‐marker activity. Female athletes showed higher device‐estimated VO_2_ max and lower exercise heart‐rate indices, accompanied by lower bioelectrical impedance‐derived estimates of fat mass, FFM, total body water, and bone mass. These contrasting patterns are compatible with recognized sex‐related variation in exercise adaptation [19, 20, 21], but do not establish sex‐specific efficacy.

Within the selected LT cohort, response patterns diverged further. Male participants showed higher FFM, skeletal muscle mass, and protein‐mass estimates, alongside continued differences in metabolic and redox‐related measures. Female participants showed lower adiposity estimates, higher device‐estimated VO_2_ max, and lower exercise heart‐rate indices, although modest increases in fasting glucose and MDA indicated that the LT pattern was not uniformly favorable. These observations may reflect differences in physiological adaptation during sustained training; however, they remain hypothesis‐generating because LT eligibility was determined after randomization and the sex‐specific subgroups were small.

Baseline descriptive comparisons identified larger standardized differences for fasting glucose and T‐AOC than for most other measured characteristics. Because the longitudinal models evaluated within‐participant change from baseline, treatment contrasts were estimated relative to each participant's starting value; nevertheless, baseline‐dependent change or regression to the mean cannot be fully excluded for these biomarkers. Across the exploratory stratified analyses, early metabolic and redox‐related patterns were followed by later differences in body‐composition estimates and field‐based cardiorespiratory measures. Such phase‐related divergence would be difficult to identify in cross‐sectional or single‐time‐point studies [6, 16, 17], although LT selection precludes direct inference of progression within the full randomized population, and the underlying biological pathways remain hypothetical. During the randomized ST phase, nominal overall differences were observed for two of the 14 gastrointestinal questionnaire items (chest discomfort and defecatory urgency), whereas the remaining items showed no clear overall differences. Given the multiple item‐level comparisons, these findings do not establish a broad gastrointestinal effect; and isolated item‐level findings in the selected LT cohort should be interpreted cautiously. The baseline‐adjusted ST sensitivity analyses further narrow the interpretation of the randomized physiological findings: the lower ExHRave contrast at Week 6 and the higher T‐AOC contrast at Week 3 were retained, whereas the Week 6 lactate and T‐AOC contrasts attenuated. These results support outcome‐ and visit‐specific interpretation rather than a uniform physiological profile and do not exclude residual effects of unmeasured baseline characteristics.

Corresponding heterogeneity was observed in gut multi‐omics data. In ST males, exploratory indirect‐effect models linked features involved in microbial one‐carbon metabolism (*gcvT*), cobamide biosynthesis (*cobT*), and Fe‐S cluster assembly (*sufB*) with lactate and antioxidant‐marker outcomes. These functions provide biologically plausible candidates at the interface between microbial metabolism, exercise, and redox biology [22, 23, 24, 31, 32], but the models do not demonstrate reduced host‐microbe substrate competition or causal mediation. In ST females, features related to cell‐wall biosynthesis (*rmlD*), phospholipid metabolism (*pgsA*), and tRNA quality control (*dtd*, *tsaC*) were associated with bioelectrical impedance‐derived body‐composition estimates and device‐estimated VO_2_ max. Their correspondence with pathways relevant to endurance and muscle physiology [8, 28, 29, 30] supports targeted follow‐up rather than a defined mechanism.

Within the selected LT cohort, exploratory male models nominated associations involving *PISD*‐linked phosphatidylethanolamine metabolism, hypoxanthine, and andrographolide. Prior work links these features to mitochondrial membrane biology [42, 43, 44], purine turnover during exercise [40, 41], and Nrf2‐related redox signaling [22, 39], providing biological context but not evidence that these pathways were activated by CPYE. Female models nominated bile acid‐, lactate‐, folate‐, histamine‐, and nucleotide‐related features, including *B. ovatus* and *folK*, in relation to device‐estimated VO_2_ max, T‐AOC, and wearable‐derived outcomes. Literature on redox and gut‐brain signaling provides context for these candidates [49, 50, 52, 53, 54, 55, 56], but the present data do not establish compensatory, autonomic, or folate‐mediated mechanisms. Collectively, the indirect‐effect models prioritize testable candidates rather than causal microbial pathways.

Beyond compositional signals, CPYE‐ and placebo‐derived networks differed in edge density, average path length, largest connected‐component ratio, and simulated node‐removal metrics. In network‐theoretic terms, higher connectivity and shorter path length are compatible with a more integrated topology and greater simulated robustness. However, because these descriptive networks were constructed from a screened feature set and included small exploratory strata, particularly the LT female subgroup, they cannot establish in vivo ecological stability, functional integration, or resistance to physiological perturbation. Microbial α‐diversity did not show consistent between‐group or temporal differences, indicating that network contrasts were not accompanied by broad changes in within‐sample diversity; neither result validates a functional or resilient ecosystem state.

Baseline physiological profile may also contribute to the observed response heterogeneity. Unsupervised clustering of pre‐intervention cardiorespiratory and body‐composition metrics identified two phenotypic clusters with 91.3% concordance with biological sex. When mixed‐effects models were refitted using these clusters instead of sex, effect directions were largely concordant across performance, metabolic, and digital‐physiology endpoints. These observations support evaluating baseline physiological profile alongside sex as a candidate stratification factor in future microbiome‐targeted trials [19, 20, 21]. Because cluster membership and sex were highly overlapping, their independent contributions cannot be distinguished in this cohort and require prospective validation in less collinear populations.

By integrating continuous wearable monitoring with shotgun metagenomics and untargeted metabolomics, this study extends earlier athlete‐microbiome and longitudinal multi‐omics work [3, 14, 15] by placing physiological and gut multi‐omics responses within a shared temporal framework. Only a limited core of features was common across strata, suggesting context‐dependent associations rather than a universal microbial signature. This pattern motivates prespecified, physiology‐informed stratification and independent validation in future trials.

The strengths of this study include its real‐world training context, longitudinal design, broad phenotyping, and integration of physiological and multi‐omics data. Several limitations nevertheless temper interpretation. First, VO_2_ max was derived from wearable algorithms during field testing rather than measured via metabolic‐cart, representing a field‐based cardiorespiratory index rather than a direct physiological endpoint. Heart rate and lactate metrics, bioelectrical impedance‐derived body composition (hydration‐sensitive), and point‐of‐care capillary glucose provide practical context but lack laboratory‐grade validation. Second, LT eligibility required ST completion and confirmed competition entry, followed by intensified training. This post‐randomization selection altered the inference population, limiting precision in the small cohort (*n* = 44) and sex strata. Although differences in selected physiological measures were smaller at Week 6 (absolute SMDs < 0.20), sensitivity analyses yielded imprecise estimates and attenuated the T‐AOC contrast, supporting cautious, hypothesis‐generating interpretation of the LT findings. Third, despite standardized testing protocols, individual training load, dietary intake, supplement use, hormonal status, and environmental conditions were not systematically quantified. Wearable monitoring captured recovery metrics but did not control these exposures; residual variation may influence physiological and sex‐stratified outcomes. Fourth, the placebo was matrix‐matched (containing galacto‐oligosaccharides and strawberry powder) rather than inert. Because low‐dose galacto‐oligosaccharides can modulate the microbiota [57], observed contrasts reflect incremental effects of the active ingredients within a shared matrix, precluding isolation of prebiotic‐specific effects. Finally, screened‐feature networks and indirect‐effect models are exploratory; they describe statistical associations rather than establishing in vivo ecological stability, temporal mediation, or causality. Prospective trials with targeted experimental perturbations are required for validation.

In conclusion, the randomized 6‐week ST phase showed CPYE‐placebo differences in selected field‐based cardiorespiratory indices and metabolic and redox‐related biomarkers, accompanied by treatment‐associated variation in gut multi‐omics profiles. The exploratory extension further suggested that response patterns may vary with intervention duration, sex, and baseline physiological profile, while network and indirect‐effect analyses identified microbial and metabolic candidates for experimental follow‐up. This study therefore provides a framework for testing physiology‐informed microbiome‐targeted nutrition rather than definitive evidence of ecosystem remodeling or causal mechanisms. Ultimately, larger prospective trials incorporating direct gas‐exchange testing, standardized performance outcomes, and targeted experimental perturbations are needed to determine the reproducibility, magnitude, and biological relevance of these findings.

## 4 Methods

### 4.1 Study Design, Ethical Oversight, and Outcome Hierarchy

We conducted a two‐stage, randomized, double‐blind, placebo‐controlled trial in collegiate endurance athletes in Inner Mongolia, China, between March and June 2023. The intervention comprised a 6‐week ST phase (Weeks 0–6) followed by a 6‐week LT extension (Weeks 6–12). Participants were randomized in a 2:1 ratio to the CPYE or a matrix‐matched placebo, with original allocation maintained during the extension. Eligibility to enter LT phase required ST completion and confirmed participation in the 12th Inner Mongolia Autonomous Region University Games; these athletes underwent intensified training during Weeks 6–12. Because LT entry was determined post‐randomization, this phase constitutes a selected cohort and was analyzed exploratorily.

The protocol was approved by the Ethics Committee of the Inner Mongolia Nutrition Society (Approval No. A202207019) prior to enrollment. Public trial registration was completed retrospectively in the Chinese Clinical Trial Registry on 21 July 2025 (ChiCTR2500106261). All procedures complied with the Declaration of Helsinki and contemporary standards for human microbiome research, and written informed consent was obtained prior to any study procedures. The pre‐enrollment protocol identified device‐estimated VO_2_ max (a wearable‐derived, field‐based cardiorespiratory index) and gastrointestinal symptoms (questionnaire‐based) as primary outcome domains and specified the intervention design and duration. However, it did not designate a single primary assessment time point, a confirmatory statistical model or test, or a multiplicity‐adjustment procedure. Secondary and supportive outcomes included exercise heart‐rate indices, post‐exercise lactate, subjective stress, body‐composition estimates, and selected serum biochemical markers. The statistical hierarchy and analysis framework are described in the Statistical analysis section.

### 4.2 Participant Recruitment and Eligibility Criteria

In March 2023, 162 collegiate athlete volunteers were assessed for eligibility. Twelve were excluded (severe gastrointestinal disease, *n* = 2; antibiotic use within the prior 4 weeks, *n* = 3; recent major surgery, *n* = 1; declined participation, *n* = 6). The remaining 150 provided written informed consent and were randomized.

Participants were recruited from competitive sports teams at Inner Mongolia Agricultural University. Eligible individuals were: (1) ≥18 years; (2) engaged in structured endurance training for ≥ 1 year; (3) no extreme or unusually high‐intensity exercise in the 14 days preceding enrollment; and (4) willingness and ability to comply with study procedures. Given that the intervention was designed to support exercise‐associated gastrointestinal tolerance, we prioritized athletes reporting a history of exercise‐associated gastrointestinal symptoms, such as abdominal pain, nausea, reflux, diarrhea, or appetite loss, or recent acute episodes (e.g., gastroenteritis, vomiting, dizziness, headache), provided they met all eligibility criteria.

Exclusion criteria included: diagnosed severe gastrointestinal disease; antibiotic use within the prior 4 weeks; *Clostridioides difficile* infection within the past 3 months; recent major surgery; known allergy to any study product component; or anticipated non‐adherence. Participants were withdrawn if they used prohibited medications or supplements, experienced a study‐limiting adverse event or sports injury, voluntarily discontinued, or were lost to follow‐up. The single ST‐phase withdrawal (day 17, unrelated sports injury) contributed baseline data only and was retained in multiple‐imputation sensitivity analyses.

### 4.3 Sample‐Size Justification

A priori sample‐size estimation was performed for the randomized 6‐week ST phase, based on the between‐group comparison of the principal continuous outcome, device‐estimated VO_2_ max. Assuming a two‐sided α of 0.05, 80% power, a 2:1 allocation ratio, and a standardized treatment effect of Cohen's *d* = 0.49 (derived from prior pilot data and literature on probiotic interventions in athletic populations), a total of 150 participants was required (CPYE, *n* = 100; placebo, *n* = 50). This calculation did not incorporate an attrition allowance, as the trial was conducted within a tightly supervised collegiate training program with anticipated high retention. The exploratory LT extension, sex‐stratified analyses, multi‐omics screening, network topology assessments, and indirect‐effect modeling were not separately powered and are presented as hypothesis‐generating.

### 4.4 Randomization, Blinding, and Intervention Administration

Randomization (2:1 CPYE:placebo) was generated using a computerized algorithm, with allocation concealment maintained via sequentially numbered, opaque, sealed containers. Investigators, participants, study coordinators, and outcome assessors remained blinded to group assignment throughout the trial.

The CPYE formulation consisted of two well‐characterized probiotic strains and a standardized yeast extract. Each 2‐g sachet delivered *Lacticaseibacillus paracasei* Zhang (4 × 10^10^ CFU) and *Bifidobacterium animalis* subsp. *lactis* ProSci‐246 (2 × 10^10^ CFU), together with 0.3 g yeast extract (polypeptides content > 70%), yielding a total label‐guaranteed viable count of 6 × 10^10^ CFU per day throughout the intervention. The yeast extract (Angel Yeast Co., Ltd., Hubei, China; compliant with GB/T 20886.2‐2021) contained 75% protein (dry‐matter basis) and 20% yeast polysaccharides. Both CPYE and placebo formulations shared an identical matrix of galacto‐oligosaccharides, strawberry powder, and maltodextrin; CPYE sachets additionally contained the two probiotic strains and yeast extract. The placebo was matched to CPYE in appearance, taste, packaging, and dosing schedule, ensuring a matrix‐matched comparison. All CPYE sachets were sourced from a single manufacturing batch, and all placebo sachets from a single, separate batch. Both products were stored at 4°C and manufactured under ISO 9001 and HALAL‐certified conditions by Jinhua Yinhe Biological Technology Co., Ltd. (Zhejiang, China).

Participants consumed one sachet daily with water, as instructed in the product leaflet. Concomitant use of antibiotics or other probiotics‐containing supplements or products was prohibited throughout the intervention. Adherence was supported through reminders from coaches and team captains during scheduled training days and monitored using routine compliance records. Based on available records, estimated adherence exceeded 95% in both groups; however, exact missed‐dose counts could not be systematically verified because Sunday intake was self‐administered without direct observation.

Participants were asked to maintain their habitual diet and lifestyle throughout the intervention; most meals were obtained from campus dining facilities, but dietary intake was not systematically recorded. Training followed a centrally organized competition‐preparation schedule of the university. During the ST phase, scheduled physical‐conditioning and sport‐specific training was conducted for approximately 2 h/day, 6 days/week. Participants entering the LT extension underwent an intensified schedule of approximately 4 h/day, divided into morning and afternoon sessions. Training schedules were coordinated and overseen by coaching staff, although individual session intensity and internal or external training load were not formally quantified. Residual variation in training exposure, dietary intake, and recovery practices may have influenced physiological and multi‐omics outcomes.

### 4.5 Clinical Assessment Schedule and Biospecimen Collection

A total of 150 athletes were enrolled and randomized (CPYE, *n* = 100; placebo, *n* = 50). During the randomized ST phase (Weeks 0–6), in‐person visits occurred at baseline (Week 0), midpoint (Week 3), and endpoint (Week 6), with standardized clinical assessments and collection of blood and fecal samples. Among ST completers, 44 participants confirmed to compete in the 12th Inner Mongolia Autonomous Region University Games entered the post‐randomization selected LT extension and underwent intensified training during Weeks 6–12, with additional visits at Weeks 8, 10, and 12. In the LT phase, blood samples were collected at Weeks 10 and 12, whereas performance testing, body‐composition analysis, and fecal sampling were performed at all three extension visits.

All visits were conducted in the morning after an overnight fast and included clinical evaluations, field‐based exercise testing, biospecimen collection, and wearable device data synchronization. All procedures were performed by trained personnel following standard operating procedures. The availability of individual phenotypic measurements and biospecimens varied by assessment domain and visit; this variation is summarized in the data‐availability matrix (Figure S2) and reflected in domain‐specific sample sizes reported throughout the results.

### 4.6 Phenotypic Assessment: Body Composition, Metabolic Indices, Field‐Based Performance, and Gastrointestinal Function

Body composition was assessed using a bioelectrical impedance analyzer (BCA‐2A, Tongfang Health Technology Co., Ltd., Beijing, China), providing estimates of BMI, PBF, total fat mass, FFM, skeletal muscle mass, bone mass, total body protein, ICW, and ECW. All abbreviations, definitions, and units of study outcomes are provided in Table S1. Fasting glucose was measured via capillary fingertip sampling using a Roche Accu‐Chek Instant Glucometer (Roche Diabetes Care GmbH, Basel, Switzerland); values are reported as whole‐blood glucose equivalents rather than laboratory plasma concentrations.

Field‐based cardiorespiratory assessment was conducted during a standardized 3000‐m run on the same outdoor 400‐m track. Participants wore Garmin Forerunner 158 sports watches (identical model and acquisition batch) throughout testing. Device‐estimated VO_2_ max, ExHRave, and ExHRmax were extracted using the manufacturer's validated algorithms. The use of a common device model and standardized protocol reduced methodological heterogeneity across participants and visits but did not provide direct measurement of oxygen consumption via metabolic cart. Device‐estimated VO_2_ max was therefore treated as a wearable‐derived, field‐based cardiorespiratory index rather than a laboratory‐validated measure of maximal oxygen uptake. Post‐exercise capillary lactate was measured immediately after test completion using a Lactate Scout 4 analyzer (EKF Diagnostics, Penarth, UK) and interpreted as a complementary indicator of post‐exercise metabolic load under identical field‐testing conditions. The 3000‐m protocol provided a common exercise‐testing context and was not treated as an independently validated time‐trial performance endpoint.

Gastrointestinal function and related symptoms were assessed using a 14‐item questionnaire (Q1‐Q14; Table S4) covering defecation frequency, stool form, upper abdominal pain, chest discomfort, acid reflux, hunger pain, nausea, borborygmi, abdominal bloating, throat discomfort, halitosis, urinary odor, defecatory urgency, and tenesmus.

### 4.7 Continuous Physiological Monitoring via Wearable Sensors

Participants wore the Garmin Forerunner 158 continuously on the non‐dominant wrist throughout the 12‐week intervention, removing the device only during activities incompatible with wear. The device recorded heart rate, sleep architecture, respiratory rate, and a proprietary, algorithmically derived stress index. Data were downloaded at predefined intervals, synchronized to a central repository, and processed through a pre‐specified quality‐control pipeline that excluded days with sensor artefacts or <8 h of valid wear time. Daily and weekly summaries, including total sleep duration, sleep efficiency, respiratory rate, RHR, and stress burden, were computed and temporally aligned with clinical, performance, and multi‐omics data streams. Stress‐category variables were expressed as the percentage of monitored time spent in each Garmin‐derived stress category, normalized to sum to 100% per day.

### 4.8 Blood Sampling and Serum Biochemical Profiling

Venous blood was drawn from the antecubital vein into standardized vacuum tubes by trained physicians. Samples were processed within 1 h of collection: centrifuged at 3000 rpm for 10 min at 4°C, after which serum was aliquoted into barcoded cryovials and stored at −80 °C. All samples underwent a single thaw cycle immediately prior to batch analysis to prevent degradation from repeated freeze‐thaw cycles.

Antioxidant capacity and oxidative stress markers were quantified using colorimetric assay kits (Beijing Boxbio Science & Technology Co., China) per the manufacturer's instructions. Total antioxidant capacity was determined using the ferric reducing antioxidant power assay. Malondialdehyde, a surrogate of lipid peroxidation, was measured via the thiobarbituric acid reactive substances assay. Glutathione peroxidase activity was quantified by monitoring the reaction of reduced glutathione with hydrogen peroxide, coupled to the reduction of 5,5’‐dithiobis‐(2‐nitrobenzoic acid), spectrophotometrically at 412 nm.

Routine serum biochemistry, including total protein, albumin, total cholesterol, high‐ and low‐density lipoprotein cholesterol, triglycerides, magnesium, phosphorus, and calcium, was analyzed on a Hitachi Automatic Analyzer 3100 (Hitachi High‐Tech, Tokyo, Japan) using manufacturer‐calibrated reagents traceable to international reference standards.

### 4.9 Fecal Sample Collection, Storage, and Processing

At each scheduled visit, participants self‐collected two fecal aliquots using sterile collection kits. The first aliquot was immediately immersed in stool DNA stabilizer (Guangdong Longsee Biomedical Co. Ltd., Guangzhou, China) for shotgun metagenomic sequencing; the second was placed in a DNA/RNA‐free cryotube for untargeted metabolomic profiling. Samples were flash‐frozen on dry ice at the collection site and transported to the laboratory within 1 h. Upon arrival, all specimens were logged into a laboratory information management system with dual verification of barcode and timestamp, then stored at −80°C until batch processing. No samples underwent thawing prior to analyses.

### 4.10 Shotgun Metagenomic Sequencing and Multi‐Kingdom Microbiome Profiling

Genomic DNA was extracted from 0.5 g of each fecal sample using the QIAamp DNA Stool Mini Kit (Qiagen, Hilden, Germany) per the manufacturer's instructions. DNA integrity was confirmed by 0.6% agarose gel electrophoresis, purity assessed by NanoDrop One spectrophotometry (OD_260/280_ = 1.8–2.0; Thermo Fisher Scientific Inc., Waltham, MA, USA), and concentration quantified using the Qubit dsDNA HS assay (≥20 ng/µl; Thermo Fisher Scientific).

Library preparation began with approximately 1 µg of high‐quality DNA, sheared to approximately 350‐bp fragments using Covaris ultrasonication (Covaris, Woburn, MA, USA). Standard Illumina library construction steps followed: end repair, A‐tailing, adapter ligation, size selection, and limited‐cycle PCR amplification. Final libraries were quantified by Qubit 2.0, validated for fragment size distribution on an Agilent 2100 Bioanalyzer, and precisely quantified by qPCR (effective concentration >3 nM). Equimolar pools of qualified libraries were sequenced on an Illumina NovaSeq 6000 platform (2 × 150 bp), yielding approximately 6 Gb of raw data per sample.

A total of 577 fecal samples (across ST and LT phases) generated an average of 43 552 302 raw reads per sample. Raw reads underwent adapter trimming and quality filtering with fastp [58] (v0.23.1), discarding reads <60 nt. Host DNA contamination was removed by aligning reads to the GRCh38 human reference genome using Bowtie2 [59] (v2.4.4); only unmapped reads were retained for microbial analysis.

Bacterial and archaeal composition was profiled using MetaPhlAn4 (v4.0.6). Functional potential was inferred with HUMAnN3 [60] (v3.6), which maps reads to gene families and reconstructs MetaCyc pathway abundances, normalized to relative abundance and copy‐number corrected.

Putative phage sequences were assembled de novo into contigs, screened using PhaBox [61] to identify circular viral genomes >3 kb. Putative phage genomes were annotated, clustered into phage operational taxonomic units at ≥95% nucleotide identity via MMseqs2 [62] (v13), and quantified by mapping quality‐controlled reads with bwa‐mem2 [63] (v2.2.1). Coverage metrics were estimated using CoverM [64], and relative abundance was expressed as reads per kilobase per million mapped reads (RPKM).

Fungal taxa were profiled by Kraken2 [65] (v2.1.2) and Bracken [66] (v2.9) against a custom gut fungal reference database built from RefSeq genomes. To minimize cross‐kingdom misclassification, all candidate fungal reads were cross‐aligned against bacterial databases; reads with significant bacterial homology were excluded from fungal quantification.

### 4.11 Untargeted Fecal Metabolomics

Fecal metabolites were profiled using a high‐resolution untargeted LC‐MS platform. Briefly, 200 mg of stool was homogenized in 600 µL of ice‐cold methanol containing 2‐chloro‐*L*‐phenylalanine (4 ppm) as an internal standard. Samples were vortexed, homogenized with stainless‐steel beads (50 Hz, 120 s), sonicated at room temperature for 10 min, and centrifuged at 12 000 rpm at 4°C for 10 min. The supernatant was filtered through a 0.22 µm polytetrafluoroethylene membrane into autosampler vials [67].

Chromatographic separation was performed on a Vanquish UHPLC system coupled to an Orbitrap Exploris 120 mass spectrometer (Thermo Fisher Scientific). Reversed‐phase chromatography used an ACQUITY UPLC HSS T3 column (2.1 × 100 mm, 1.8 µm; Waters, Milford, MA, USA) maintained at 40°C, with a flow rate of 0.30 mL/min and 2‐µL injection volume. Mobile phases consisted of (A) water and (B) acetonitrile, both containing 0.1% formic acid for positive ion mode or 5 mM ammonium formate for negative ion mode. A linear gradient eluted metabolites from polar to nonpolar compounds. Mass spectrometry was performed in both positive and negative electrospray ionization modes, with spray voltages of +3.5/–2.5 kV and capillary temperature set to 325°C. Full‐scan MS1 spectra were acquired from *m/z* 100–1000 at 60 000 resolutions. Data‐dependent MS2 spectra were acquired using higher‐energy collisional dissociation at a normalized collision energy of approximately 30%, with 15 000 resolution and dynamic exclusion enabled to minimize redundant precursor selection.

Raw data files were converted to open mzML format using ProteoWizard (v3.0.8789) and processed with XCMS (v3.12.0) for peak detection, retention time correction, and feature alignment across samples. Batch effects and signal drift were corrected using support vector regression based on internal standard and quality control sample trends. Features exhibiting >50% missing values across samples or poor technical reproducibility were excluded.

Metabolite annotation combined in‐house spectral libraries, public databases (e.g., HMDB, METLIN), and the metDNA pipeline. Data were median‐normalized, log‐transformed, and auto‐scaled before statistical analysis. Differentially abundant metabolites were identified using MetaboAnalyst 6.0 [68], with significance defined by a variable importance in projection score > 1 and a *p* < 0.05.

### 4.12 Statistical Analysis

All statistical analyses and visualizations were performed in R (v4.5.0), using two‐sided tests. The pre‐enrollment protocol did not specify a confirmatory statistical test, designate either Week 3 or Week 6 as a confirmatory primary time point, or define a study‐wide multiplicity‐adjustment procedure across the two primary outcome domains.

Accordingly, visit‐specific LMM estimates, Wilcoxon *P* values, and the separate permutation‐based trajectory tests are reported as nominal or supportive rather than prospectively confirmatory. Week 6 estimates and 95% CIs are presented as the end‐of‐ST contrasts. The randomized ST total‐cohort analyses provide the principal intervention comparisons, secondary physiological outcomes are supportive, and all LT, sex‐stratified, cluster‐stratified, network, and indirect‐effect analyses are exploratory and hypothesis‐generating. Multiple‐testing correction was applied only where explicitly stated for the corresponding analysis.

Baseline characteristics of the 150 randomized participants were summarized by treatment group as mean ± SD for continuous variables and *n* (%) for categorical variables. Between‐group differences were quantified using absolute SMDs to describe balance without relying on significance testing of randomization. Baseline summaries used observed data without imputation [69].

To account for inter‐individual variability, we analyzed change‐from‐baseline (Δ = follow‐up minus baseline) for all clinical, performance, and wearable‐derived outcomes. LMMs with participant modeled as a random intercept were fitted separately for the ST and LT phases to estimate CPYE‐placebo differences in longitudinal trajectories. Models were adjusted for baseline age, BMI, PBF, and ethnicity [70]. Global tests of treatment‐by‐time interactions were performed to assess differential trajectories using custom scripts. Estimates from the LT extension were treated as exploratory due to post‐randomization cohort selection. For the descriptive violin plots, visit‐specific CPYE‐placebo comparisons were evaluated using nominal two‐sided Wilcoxon rank‐sum tests; these tests were distinct from the LMM analyses and the permutation‐based trajectory tests.

To assess the impact of the single ST phase withdrawal, we conducted intention‐to‐treat sensitivity analyses including all 150 randomized participants. The withdrawn participant contributed baseline data only; missing Week 3 and Week 6 outcomes were multiply imputed under a missing‐at‐random assumption using predictive mean matching (50 imputations). The imputation model included treatment allocation, baseline outcome value, sex, age, BMI, PBF, ethnicity, and available longitudinal measurements. LMMs were fitted to each imputed dataset, and CPYE‐placebo contrasts were pooled using Rubin's rules. For continuous outcomes, delta‐adjusted sensitivity analyses shifted imputed follow‐up values by −2, −1, +1, or +2 pooled SDs of observed change scores. Bounded gastrointestinal questionnaire items were assessed under the missing‐at‐random model without delta shifting. These analyses evaluated the robustness of ST estimates to alternative handling of the single missing record.

To evaluate sensitivity to baseline imbalance during the randomized ST phase, we fitted supportive baseline‐adjusted LMMs for device‐estimated VO_2_ max, post‐exercise lactate, fasting glucose, ExHRave, and T‐AOC. Raw Week 3 and Week 6 follow‐up values were modeled with fixed effects for treatment, categorical visit, treatment‐by‐visit interaction, the corresponding baseline outcome and its interaction with visit, baseline age, BMI, PBF, and ethnicity, with participant as a random intercept. Baseline outcome values were thus used as covariates rather than incorporated into a change‐score calculation. Models were fitted in the observed‐data cohort (*n* = 149) and repeated across the 50 multiple‐imputation intention‐to‐treat datasets (*n* = 150), with visit‐specific estimates combined using Rubin's rules. These sensitivity analyses reported nominal two‐sided *P* values, with no additional multiplicity adjustment.

To characterize observable post‐randomization selection into the LT extension, we compared LT entrants with ST completers who did not enter LT across original treatment allocation, sex, age, BMI, and eight key physiological measures at baseline and Week 6, using signed SMDs (Table S2, Panel A). Parsimonious LT sensitivity analyses were fitted to model raw post‐Week 6 outcomes with fixed effects for treatment, categorical time, treatment‐by‐time interaction, the corresponding Week 6 value, and sex, and a participant‐level random intercept (Table S2, Panel B). Week 6 values served as baseline covariates for the extension and were not modeled as repeated outcomes; sex was included a priori given its differential distribution between entrants and non‐entrants. Given the small LT cohort (*n* = 44), no additional covariates were included, and missing values were not imputed. These exploratory analyses assess selection bias and model sensitivity.

To explore baseline physiological heterogeneity, we applied unsupervised hierarchical clustering to *z*‐scored baseline values of key phenotypes (device‐estimated VO_2_ max, heart‐rate metrics, adiposity, and FFM) using Euclidean distance and Ward's D2 linkage. The optimal number of clusters was determined by maximizing average silhouette width, yielding a two‐cluster solution (Cluster 1 vs. Cluster 2). These clusters were incorporated as strata in sensitivity LMMs to evaluate whether CPYE‐placebo contrasts varied by baseline physiological profile.

For microbiome and metabolome data, α‐diversity was quantified using Shannon and Simpson indices and observed species richness. β‐diversity was assessed using Aitchison distance to account for compositional structure. The contribution of intervention group, sex, time, and covariates to overall variance was evaluated by PERMANOVA (vegan R package; 999 permutations). To account for repeated measures and temporal structure, we applied CTF across time points and computed Aitchison and Euclidean distances in the first three CTF components for robustness checks [71]. Differential abundance testing was performed using MaAsLin2 [72], with treatment, time, age, and BMI as fixed effects and participant as a random intercept. Features detected in ≥30% of samples were retained. Within each stratum, *p* values were adjusted using the BenjaminiHochberg procedure. A false discovery rate (*q*‐value) threshold of <0.25 was used as an exploratory screening criterion to prioritize features for downstream descriptive network and exploratory indirect‐effect analyses, not as confirmatory discoveries. Multi‐omics co‐occurrence networks were constructed separately for CPYE and placebo groups within each phase and sex stratum, using Spearman correlations on centered log‐ratio‐transformed abundances of the 823 candidate features. Networks were evaluated for topology (edge count, density, average path length, largest connected component ratio) and normalized edge strength heterogeneity. Robustness to perturbation was evaluated by simulating targeted and random node removal, yielding robustness to targeted removal (*R*target) and random removal (*R*random) metrics [73].

Networks were visualized and annotated in Cytoscape [74] (v3.10.3). Exploratory indirect‐effect analyses were conducted to identify candidate microbial and metabolic features statistically associated with CPYE exposure and host outcomes [75]. High‐influence network nodes (degree ≥ 3 and normalized edge strength heterogeneity ≥ 0.8) were selected as candidate features. Within each time and sex stratum, hierarchical linear mediation models were fitted to estimate indirect effects for outcomes including device‐estimated VO_2_ max, lactate, fasting glucose, antioxidant markers, body‐composition estimates, and wearable‐derived heart‐rate and sleep metrics. Models were adjusted for age, BMI, and ethnicity, with participant as a random intercept. These analyses were used to nominate candidate statistical pathways and were not interpreted as evidence of causal mediation.

Together, these analytical approaches were designed to characterize sex‐ and phase‐specific associations and prioritize candidate pathways for future experimental validation; they do not establish causal biological mechanisms or confirmatory efficacy.

## Author Contributions

W.Y.Z. and Z.H.S. conceptualized the study and designed the overall research framework. M.Z. and K.Y.L. conducted the experimental work and data collection. L.G., Y.R.L., and G.L.Z. coordinated athlete recruitment and oversaw daily training schedules, load management, and performance testing. M.Z. developed the analytical methodology and performed all statistical and multi‐omics analyses, with methodological support from Z.S. M.Z. and Z.S. prepared the original draft of the article. M.Z., Z.S., and L.Y.K. reviewed and extensively revised the article for intellectual content. W.Y.Z. and Z.H.S. supervised the study and provided critical oversight. All authors read and approved the final article.

## Conflicts of Interest

The authors declare no conflicts of interest.

## Supporting information




**Supporting File 1**: advs77664‐sup‐0001‐SuppMat.pdf.

**Supporting File 2**: advs77664‐sup‐0002‐SuppMat.xlsx.

## Data Availability

Raw shotgun metagenomic sequencing data generated in this study have been deposited in the Genome Sequence Archive at the National Genomics Data Center, China National Center for Bioinformation/Beijing Institute of Genomics, Chinese Academy of Sciences, under accession number PRJCA054940. Processed source data underlying all figures and analyses, together with custom R scripts, have been deposited in a curated reproducibility package on figshare (https://doi.org/10.6084/m9.figshare.32043930).
