# Supplementary material for: Sex‐ and Time‐Resolved Physiological and Gut Ecosystem Responses to a Food‐Grade Microbiome‐Targeted Intervention in Collegiate Endurance Athletes
Source: Adv Sci (Weinh). 2026 Oct 12:e77664. Online ahead of print. doi: 10.1002/advs.77664 (PMC13656827; doi:10.1002/advs.77664)
Supplement: Supplementary file 1 [file ADVS-9999-e77664-s001.pdf]

Sex- and Time-Resolved Physiological and Gut Ecosystem Responses to a Food-Grade Microbiome-Targeted Intervention in Collegiate Endurance Athletes

Supplementary Materials

Supplemental Figures

Figure S1. CONSORT participant flow diagram

Figure S2. Overview of multimodal data availability across trial phases

Figure S3. Exploratory baseline physiological clustering and sex-stratified response patterns

Figure S4. PERMANOVA associations between covariates and multi-omics variation

Figure S5. Alpha-diversity metrics by treatment, phase, and sex

Supplemental Figures

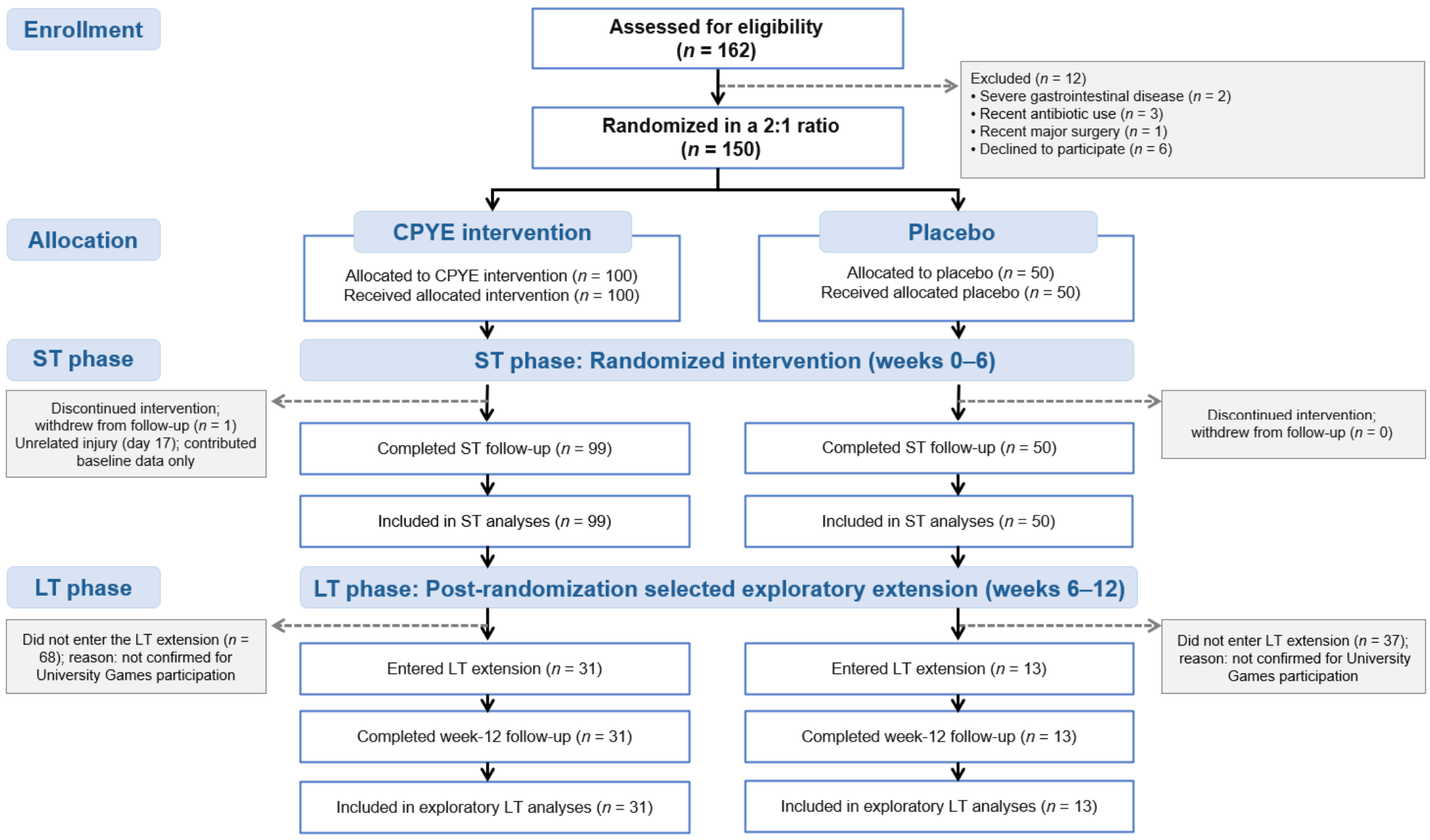

Figure S1. CONSORT participant flow diagram. ST = short-term (6-week randomized phase); LT = long-term (6-week selected extension). Of 162 collegiate athlete volunteers assessed for eligibility, 12 were excluded (severe gastrointestinal disease, *n* = 2; recent antibiotic use, *n* = 3; major surgery, *n* = 1; refusal, *n* = 6). The remaining 150 participants were randomized 2:1 to the combined probiotics-yeast extract intervention (CPYE; *n* = 100) or placebo (*n* = 50). One CPYE participant withdrew on day 17 due to an unrelated sports injury and contributed baseline data only; 149 completed ST phase. Primary ST analyses used the observed-data cohort (*n* = 149; CPYE, *n* = 99; placebo, *n* = 50); a multiple-imputation intention-to-treat sensitivity analysis included all 150 randomized participants. Among ST completers, LT eligibility required confirmed participation in the 12th Inner Mongolia Autonomous Region University Games. Forty-four participants retained original assignments, entered the LT extension under intensified training, and completed week-12 follow-up (CPYE, *n* = 31; placebo, *n* = 13); 105 ST completers did not enter LT due to non-participation in the University Games (CPYE, *n* = 68; placebo, *n* = 37). Because LT eligibility was determined post-randomization, the LT cohort represents a selected subset, and all LT and LT-by-sex analyses are presented as exploratory and hypothesis-generating. Availability of individual phenotypic measurements and biospecimens varied by domain and is summarized in **Figure S2**.

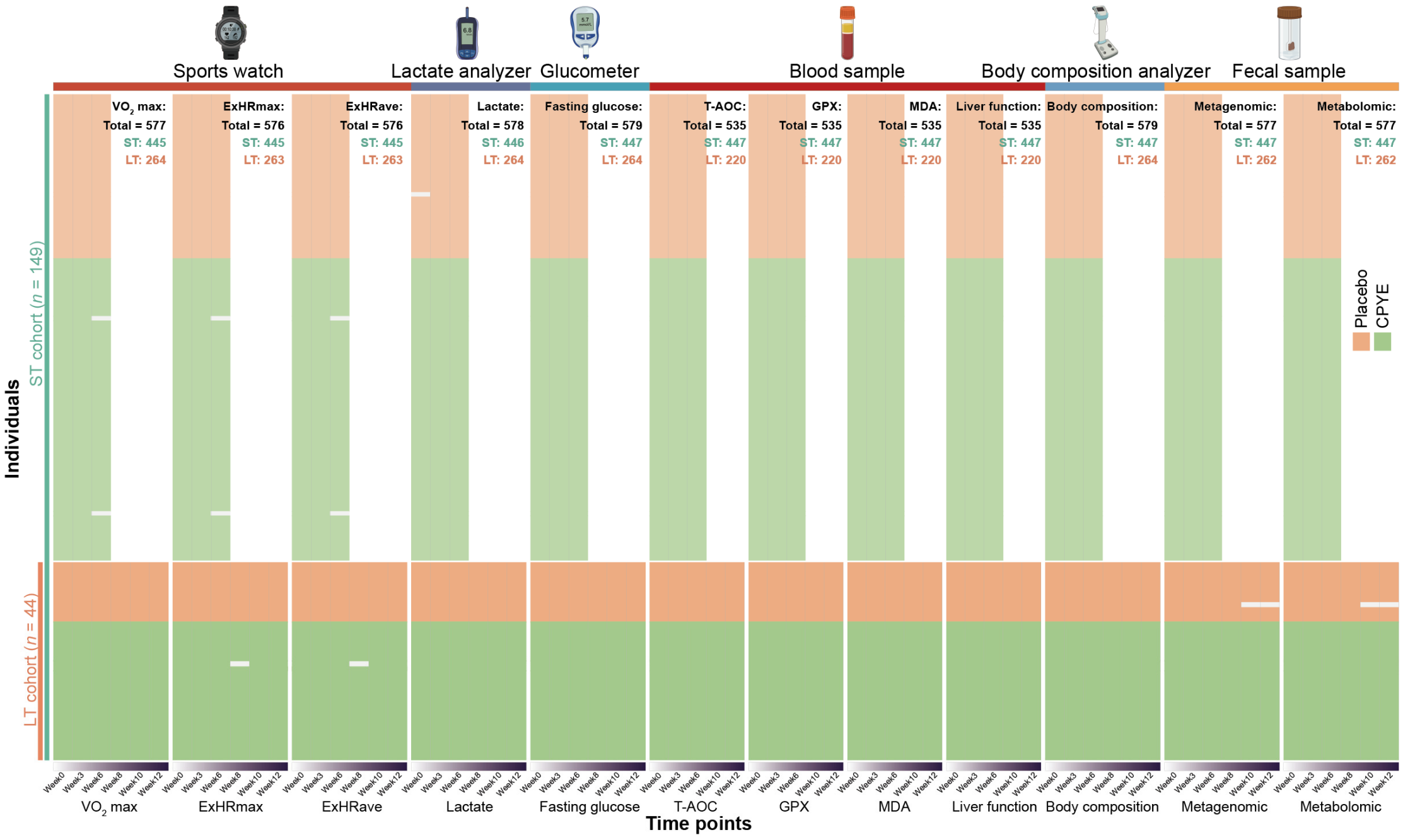

Figure S2. Overview of multimodal data availability across trial phases. ST = short-term (6-week randomized phase); LT = long-term (6-week selected extension). The availability matrix summarizes participant-level measurements and biospecimens retained for each assessment domain and study visit. Rows represent individual participants ordered by original randomized assignment; columns represent scheduled time points. Color coding: green = CPYE, orange = placebo, white = missing or uncollected observation. The upper block shows the 149 ST completers with post-baseline data (CPYE, *n* = 99; placebo, *n* = 50). The participant who withdrew after baseline is not displayed in this availability matrix. The lower block shows the 44 participants who entered the post-randomization selected LT extension (CPYE, *n* = 31; placebo, *n* = 13) with complete longitudinal records from weeks 6-12. LT-cohort counts include these participants' weeks 0-6 observations, which also appear in the ST cohort; ST and LT counts therefore overlap. The "Total" value above each domain represents the number of unique participant-visit observations or biospecimens available across the full dataset. Assessment domains include: device-estimated maximal oxygen uptake (VO_2 max), maximal and average exercise heart rate (ExHRmax and ExHRave), post-exercise blood lactate (lactate), fasting glucose, serum antioxidant markers (total antioxidant capacity, T-AOC; glutathione peroxidase, GPX; malondialdehyde, MDA), liver function, body composition, and fecal multi-omics. The horizontal axis indicates measurement timing for each variable; the total number of valid observations per variable is displayed at the top of each column. White cells denote missing or uncollected observations for the corresponding participant-variable pair. Additional abbreviations are defined in **Table S1**.

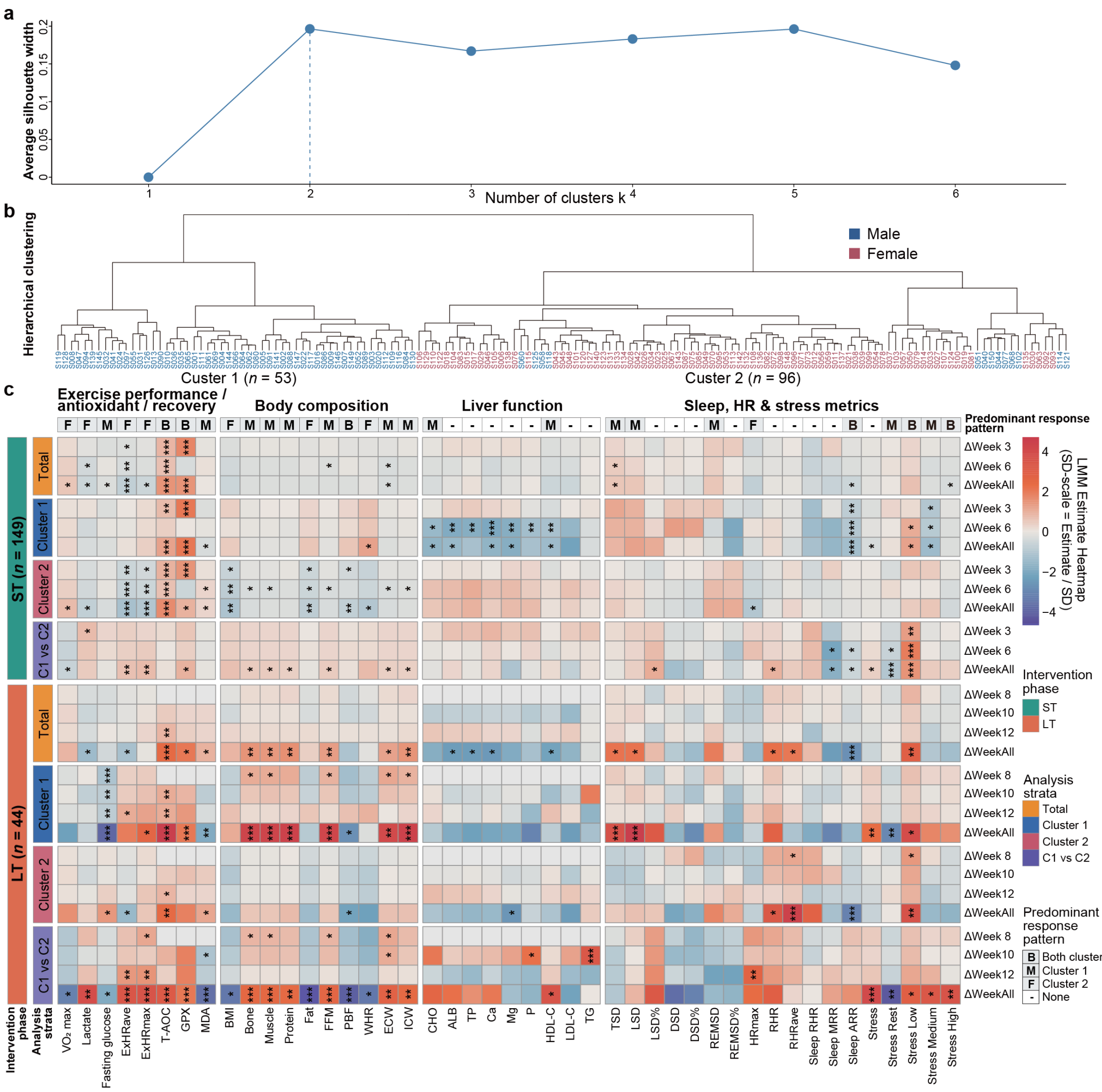

Figure S3. Exploratory baseline physiological clustering and sex-stratified response patterns. a, **Cluster selection.** Average silhouette width across candidate cluster numbers; $k = 2$ was selected as optimal. **b**, **Dendrogram and sex concordance.** Hierarchical clustering of 149 participants based on Euclidean distances of z-scored baseline phenotypes. Terminal nodes are colored by sex (blue = male, red = female). Two clusters emerged: Cluster 1 (all male, *n* = 53) and Cluster 2 (83 female, 13 male, *n* = 96), yielding 91.3% concordance between sex and cluster assignment. **c**, **Heatmap of CPYE-placebo contrasts.** Rows represent linear mixed-model (LMM) contrasts for Total, Cluster 1, Cluster 2, and Cluster 1 vs. Cluster 2 analyses across short-term (ST; 6-week randomized phase) and long-term (LT; 6-week selected extension) phases. Columns correspond to field-based cardiorespiratory and redox-related measures, body-composition estimates, serum biochemistry, and wearable-derived sleep and stress metrics. Colors indicate standardized, covariate-adjusted LMM coefficients for CPYE vs. placebo (red = positive contrast, blue = negative contrast). Response-pattern symbols above columns denote the predominant nominal pattern: both clusters, Cluster

1, Cluster 2, or no predominant pattern. Sample sizes: ST total *n* = 149 (CPYE 99, placebo 50); Cluster 1 *n* = 53 (CPYE 33, placebo 20); Cluster 2 *n* = 96 (CPYE 66, placebo 30). LT extension *n* = 44 (CPYE 31, placebo 13); Cluster 1 *n* = 10 (CPYE 4, placebo 6); Cluster 2 *n* = 34 (CPYE 27, placebo 7). Asterisks denote statistically significant CPYE-placebo contrasts (*P < 0.05, **P < 0.01, ***P < 0.001; two-tailed Wald test from LMM). Because cluster membership substantially overlapped with sex, these analyses do not separate the independent contributions of baseline physiology and biological sex. All cluster-stratified and LT-phase results are presented as exploratory and hypothesis-generating. Abbreviations are defined in **Table S1.**

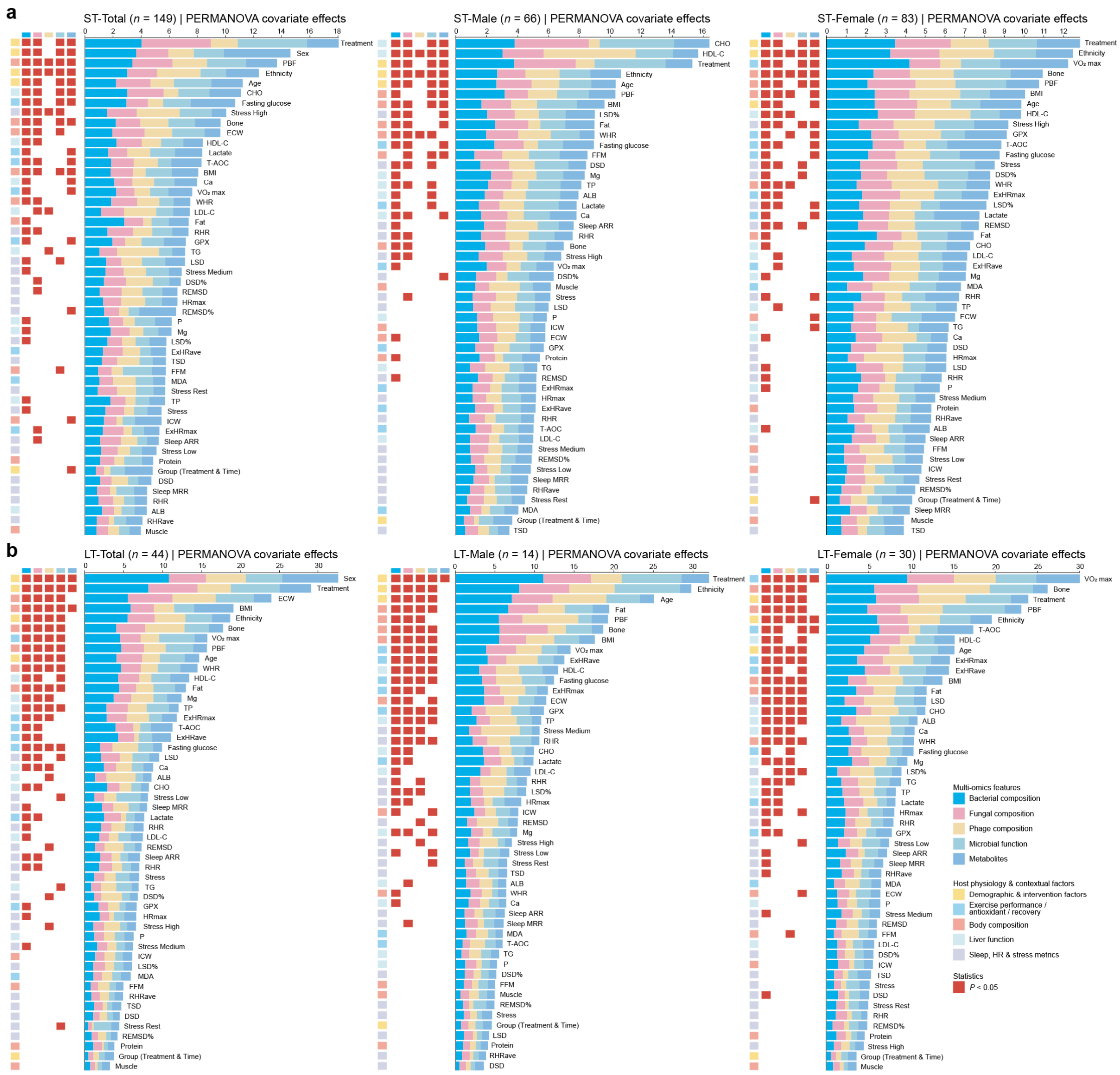

Figure S4. PERMANOVA associations between covariates and multi-omics variation. Horizontal bar plots show pseudo-*F* statistics for associations between covariates and variation in Aitchison distances across five omics layers: bacterial composition, fungal composition, phage composition, microbial functional profiles, and fecal metabolite profiles. **a, Short-term (ST) phase.** Randomized 6-week intervention (*n* = 149 with post-baseline samples; CPYE, *n* = 99; placebo, *n* = 50). **b, Long-term (LT) phase.** Post-randomization selected extension (*n* = 44), stratified by sex (males, *n* = 14; females, *n* = 30). Covariates, including microbiome, host, or experimental factors, are grouped and color-coded. Red squares indicate statistically significant PERMANOVA associations ($P < 0.05$, 999 permutations). Additional abbreviations are defined in **Table S1**.

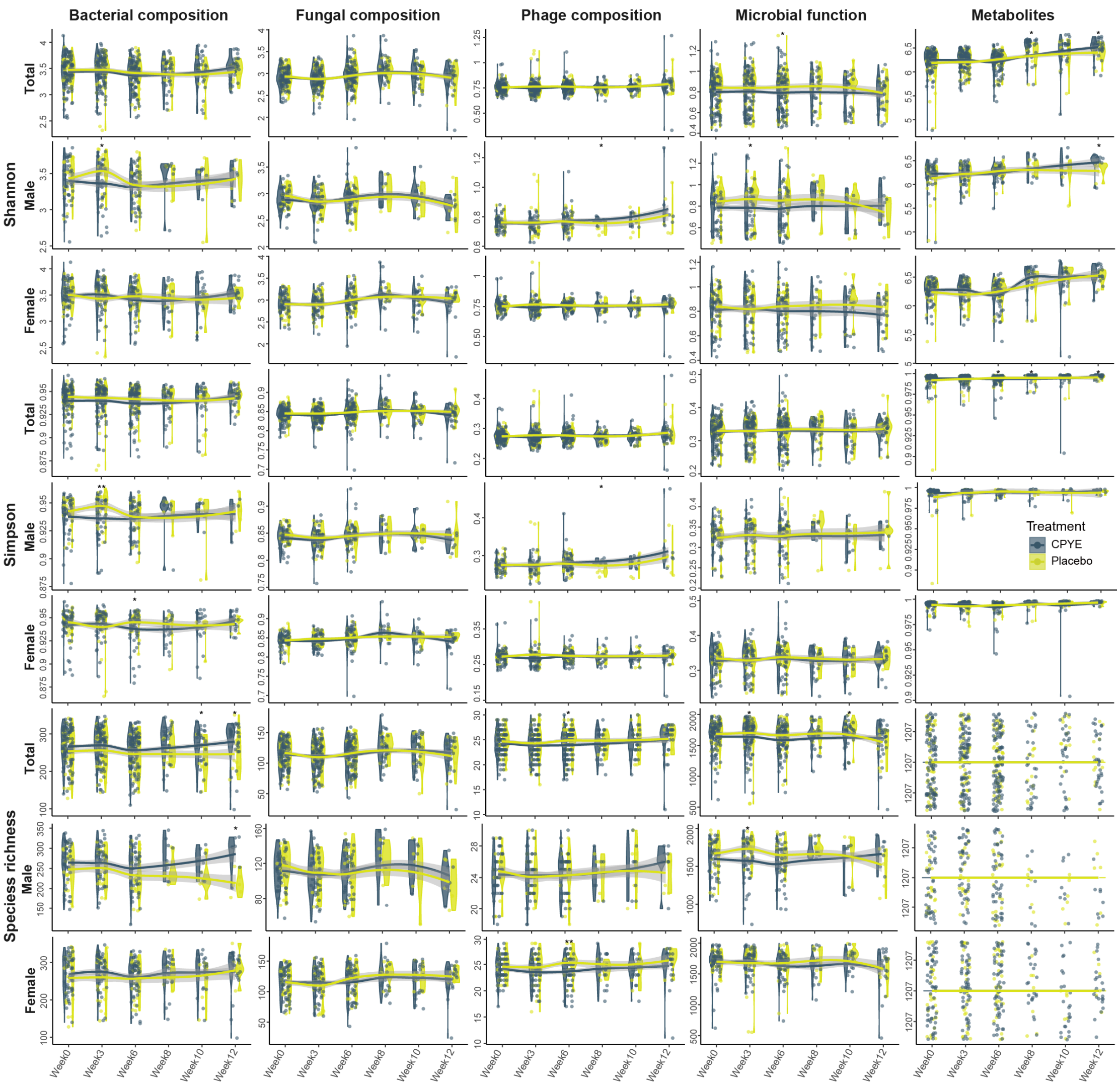

Figure S5. Alpha-diversity metrics by treatment, phase, and sex. Shannon, Simpson, and richness indices for bacterial, fungal, phage, functional, and metabolite profiles. Data shown for short-term (ST) phase (n = 149; CPYE, n = 99; placebo, n = 50) and long-term (LT) phase (n = 44), stratified by sex. Lines represent smoothed group-level trajectories; shaded bands indicate 95% confidence intervals. Color encoding: CPYE = blue, placebo = yellow. No consistent directional differences between CPYE and placebo were observed across feature layers or time points ($P > 0.05$, two-tailed Wilcoxon rank-sum test). These results indicate that the compositional differences were not accompanied by systematic shifts in the displayed diversity metrics; they do not establish ecological benefit or stability.
